# The global landscape and research trend of lymphangiogenesis in breast cancer: a bibliometric analysis and visualization

**DOI:** 10.3389/fonc.2024.1337124

**Published:** 2024-03-14

**Authors:** Liuyan Xu, Xuan Wang, Beibei Wang, Bingxin Meng, Xiaohua Pei

**Affiliations:** ^1^ Department of Breast Surgery, The Third Affiliated Hospital of Beijing University of Chinese Medicine, Beijing, China; ^2^ Department of Breast Surgery, The First Affiliated Hospital of Henan University of Chinese Medicine, Zhengzhou, China; ^3^ Department of Breast Surgery, The Xiamen Hospital of Beijing University of Chinese Medicine, Xiamen, China

**Keywords:** bibliometric analysis, breast cancer, lymphangiogenesis, visualization, Citespace, VOSviewer

## Abstract

**Background:**

Breast cancer persists as a major public health issue on a global scale. Lymphangiogenesis is an indispensable element in the promotion of breast cancer metastasis. Inhibiting the metastasis of breast cancer can be accomplished through targeting lymphangiogenesis. The purpose of this study was to examine research trends, major topics, and development directions of lymphangiogenesis in breast cancer through a bibliometric analysis, which may serve as a reference for future research and clinical practice.

**Methods:**

English publications with article type article or review about lymphangiogenesis in breast cancer from inception to September 30, 2023, retrieved from the Web of Science Core Collection Database (WOSCC), and VOSviewer, CiteSpace, and Microsoft Excel were applied for bibliometric study.

**Results:**

In this paper, a total of 369 articles and reviews were included. The 369 papers were written by 2120 authors from 553 organizations across 42 countries, published in 199 journals, and cited 12458 references from 1801 journals up to September 30, 2023. Moreover, the annual publications had a rising trajectory between 2004 to 2014 but declined from 2015. The US was the leading nation in publications and citations. Meanwhile, academics Mousumi Majumder and Peeyush Lala had the highest cumulative number of publications. Based on the number of publications/citations, Cancer Research was the most influential journal. The most cited paper was “Lymphangiogenesis: Molecular Mechanisms and Future Promise” by Tuomas Tammela, published in the Journal of Cell. Additionally, keywords frequency analysis demonstrated that “lymphangiogenesis,” “breast cancer,” “VEGF-C,” “angiogenesis,” and “metastasis” were the most frequent keywords, and the newly emergent topics could be represented by “tumor microenvironment,” “metastasis,” “stem-cell,” “triple-negative breast cancer,” and “blood vessels.”

**Conclusions:**

Currently, there is a strong research basis for lymphangiogenesis in breast cancer. The core research team was primarily situated in the US. Investigating the mechanism of lymphangiogenesis in breast cancer will always remain a highly discussed topic. In particular, it was essential to emphasize the relationship between lymphangiogenesis and tumor microenvironment, stem cells, triple-negative breast cancer, and metastasis, which could be the frontiers.

## 1 Introduction

Breast cancer is the most frequently diagnosed cancer, the predominant cause of mortality in women, and has the highest incidence of malignant tumors. The percentage of breast cancer patients who may develop metastases post-diagnosis and primary tumor treatment is estimated to be between 20-30%, and around 90% of cancer-related deaths are attributed to metastasis (1). The 5-year overall survival (OS) rate of breast cancer patients without metastasis is greater than 80% (2). Nevertheless, distant metastasis can cause a remarkable decline in the rate, resulting in only about 25% (3). Therefore, systematic and deepened research on the molecular heterogeneity of metastatic breast cancer, which is a potential cause of a significant number of therapeutic failures, would afford the ability to explore more effective metastasis-targeting agents and improve patient prognosis. Blood vessels and lymphatic vessels are the main routes of tumor metastasis. However, in many solid tumors, the lymphatic system is the major escape route for cancer cells. Lymph nodes in the drainage area are usually the initial site of metastasis, especially for breast cancer.

Due to the rich mammary lymphatic network, cytokines such as chemokines, vascular endothelial growth factors (VEGF), and other immune factors, breast cancer cells tend to metastasize to the lymph nodes located in the drainage area. These factors not only cause tumor lymphangiogenesis but also facilitate the transfer of cancer cells to certain organs through the lymphatic system (4, 5). Lymphangiogenesis is deemed to be a vital step for cancer cells to metastasize through lymphatic vessels (6, 7). Research on cancer animal models and human tumor clinicopathological analysis also highlights that increased intratumoral and peritumoral lymphatic vessels are directly associated with cancer metastasis, and lymphangiogenesis is related to the poor clinical prognosis of breast cancer (5, 7). Recently, although anti-tumor angiogenesis drugs such as bevacizumab have had limited success in improving the OS of patients in clinical trials (8), this has prompted researchers to recognize that only targeting tumor angiogenesis cannot effectively impede breast cancer metastasis; rather, targeting lymphatic vessels and lymphangiogenesis may be a successful strategy to contain metastasis.

With the proliferation of literature, it is a challenge for researchers to accurately and promptly capture the research topics and trends in the academic realm without any prior knowledge. A quantitative and qualitative method of analysis of literature, bibliometrics utilizes statistical analysis methods to assess the structure, quantitative relationships, and changing patterns of relevant data in the literature. It can illustrate the current research progress, hotspots, and trends in a particular field from multiple perspectives, as well as indicate future development directions (9). Hence, it is imperative to acquire data and information from various pertinent publications on lymphangiogenesis in breast cancer in recent years in order to assist researchers in finding valuable and referential literature information on the topic. However, we have not found any bibliometric research in this field. Therefore, we executed this research to (a) help clinicians and researchers distinguish outstanding authors, countries, and journals with academic activity in this field, (b) summarize and evaluate the research achievements and trends, and (c) generate an effective reference for further research in the field of lymphangiogenesis in breast cancer.

## 2 Materials and methods

### 2.1 Data sources and collection

The Web of Science Core Collection (WOSCC) (http://apps.webofknowledge.com) database is the most commonly used and widely accepted database in scientific, among which the Science Citation Index Expanded (SCI-E) is regarded as the most suitable database for bibliometric analysis (10). In this study, literature on lymphangiogenesis in breast cancer was systematically retrieved from the SCI-E of WOSCC. “Breast cancer” and “lymphangiogenesis” were used as the search terms in the Medical Subject Headings (MeSH) database (https://www.ncbi.nlm.nih.gov/mesh). Ultimately, the full search strategy for obtaining data is as follows. [TS=(Breast Neoplasm OR Neoplasm, Breast OR Breast Tumors OR Breast Tumor OR Tumor, Breast OR Tumors, Breast OR Neoplasms, Breast OR Breast Cancer OR Cancer, Breast OR Mammary Cancer OR Cancer, Mammary OR Cancers, Mammary OR Mammary Cancers OR Malignant Neoplasm of Breast OR Breast Malignant Neoplasm OR Breast Malignant Neoplasms OR Malignant Tumor of Breast OR Breast Malignant Tumor OR Breast Malignant Tumors OR Cancer of Breast OR Cancer of the Breast OR Mammary Carcinoma, Human OR Carcinoma, Human Mammary OR Carcinomas, Human Mammary OR Human Mammary Carcinomas OR Mammary Carcinomas, Human OR Human Mammary Carcinoma OR Mammary Neoplasms, Human OR Human Mammary Neoplasm OR Human Mammary Neoplasms OR Neoplasm, Human Mammary OR Neoplasms, Human Mammary OR Mammary Neoplasm, Human OR Breast Carcinoma OR Breast Carcinomas OR Carcinoma, Breast OR Carcinomas, Breast)] AND TS=(Lymphangiogenesis OR Lymphangiogeneses), and set the time span from inception to September 30st 2023, the article type as “Article” or “Review”, and the language as “English”. The final retrieval execution time was October 10, 2023. Two researchers independently conducted the retrieval. During the retrieval process, disagreements were resolved by consulting a third colleague or the entire academic team. There were 1201 documents generated in this field. Following that, a manual review was conducted to examine the contents of each article to eliminate irrelevant publications that were not relevant to this study. Eventually, after excluding 823 articles that deviate significantly from the main topic, 369 articles were ultimately included, including 289 reviews and 70 articles. The 369 papers used in this study were from 2120 authors from 553 organizations in 42 countries, published in 199 journals, and cited 12458 references from 1801 journals up to September 30st 2023. All data used in this work were downloaded from a public database; therefore, no ethics approval or informed consent was required. The above processes were carried out under the guidance of breast cancer experts and bibliometrics experts.

### 2.2 Data analysis

We obtained a complete record of all the literature that satisfied these inclusion criteria from the WOSCC, including the titles, abstracts, countries, institutions, journals, authors, cited references, and keywords, all of which were downloaded as a txt file and imported into VOSviewer R1.6.19 and CiteSpace R6.2.2. After using VOSviewer and CiteSpace to analyze these documents, 369 articles were subjected to visualization analysis. VOSviewer was applied to analyze the targeted files and exported the top-cited or productive countries/regions, authors, journals, references, keywords. Microsoft Excel 2019 (https://www.microsoft.com/, accessed November 9, 2021). was applied to create line charts and tables. At the same time, the 2022 version of Impact factor (IF), Journal Citation Reports (JCR) quartile and H-index as important indicators to measure the scientific value of research, were also included in the analysis. Impact factor (IF) is a method of quantitatively evaluating the importance of absolute or total citation ratio. Journal Citation Reports (JCR) 2022 was used to obtain the impact factor (IF) and quartile of a journal category (11)(https://jcr.clarivate.com/jcr/browse-journals). The H-index, which indicates that an academic journal or scholar/country/region has published H papers, each of which was cited at least H times, was used to evaluate the scientific impact of an author or country (12)(https://www.scimagojr.com/journalrank.php).

Bradford’s law of concentrations was applied to the journals, fragmented into thirds of articles, avoiding the exponential decrease in decreasing performance by expanding the search of references in scientific journals peripheral to the topic under study (13). Bradford’s law proposed that each type of sci-tech periodical could be placed under a certain discipline classification. If the sci-tech journal is arranged in descending order based on the number of scholarly papers published in a particular specialty, then it can be categorized into core area, related area, and non-related area. The number of articles in every district is roughly equal, and the number of journals in the core area, related area, and non-related area becomes the relationship of 1: n : $n^{2}$ during a certain period (14). Lotka’s law states that an author who publishes n papers is one $n^{2}$ of an author who publishes 1 paper, and that about 60.79% of authors publish 1 paper in a field, unveils the relationship between the author and the quantity of papers published and outlines the frequency distribution pattern of scientific productivity (15). Price’s Law is a measure of the distribution pattern of authors in various research fields of literature, states that for a research field, half of the papers are written by a group of productive authors whose number is equal to the square root of the total number of authors (16). In the formula, signifies the number of authors who have written x  papers, $I=n_{max}\;$ is the number of papers published by the most productive authors in the field (calculated through VOSviewer generated a value of 10), N  is the total number of authors (calculated by VOSviewer was 2120), and m is the minimum number of papers published by the core authors.


$$\sum_{m+1}^{I}n\left(x\right)=\sqrt{N}$$

### 2.3 Bibliometric analysis and visualization software

VOSviewer (https://www.vosviewer.com/, Version 1.6.19, accessed May 10, 2023) and CitesSpace (https://citespace.podia.com/ download,Version 6.2.2, accessed May 10, 2023) are bibliometric softwares and both pay special attention to the graphical representation of bibliometric maps. VOSviewer is a widely-used software in bibliometric analysis created by the Science and Technology Research Center of Leiden University (17). VOSviewer applies probabilistic-based data normalization methods to produce bibliometric maps in fields such as keywords, countries, and authors, with prominent features of easy mapping and beautiful images. In this study, VOSviewer and Microsoft Excel were applied to analyze and map the distribution of publications, countries, authors, journals, keywords and citations of the retrieved 369 articles, and constructed overlay map of keywords. CiteSpace is a free Java application that can visualize and analyze scientific literature trends and patterns. It was developed by Professor Chaomei Chen in 2004 and has become a research tool for studying research trends in a certain field by presenting the structure, patterns, and distribution of scientific knowledge and providing a comprehensive overview of research status. In this study, CiteSpace was used for extraction of the annual distribution information of articles and burst word analysis (18).

## 3 Results

### 3.1 Analysis of global publishing trends

**Figure 1:**
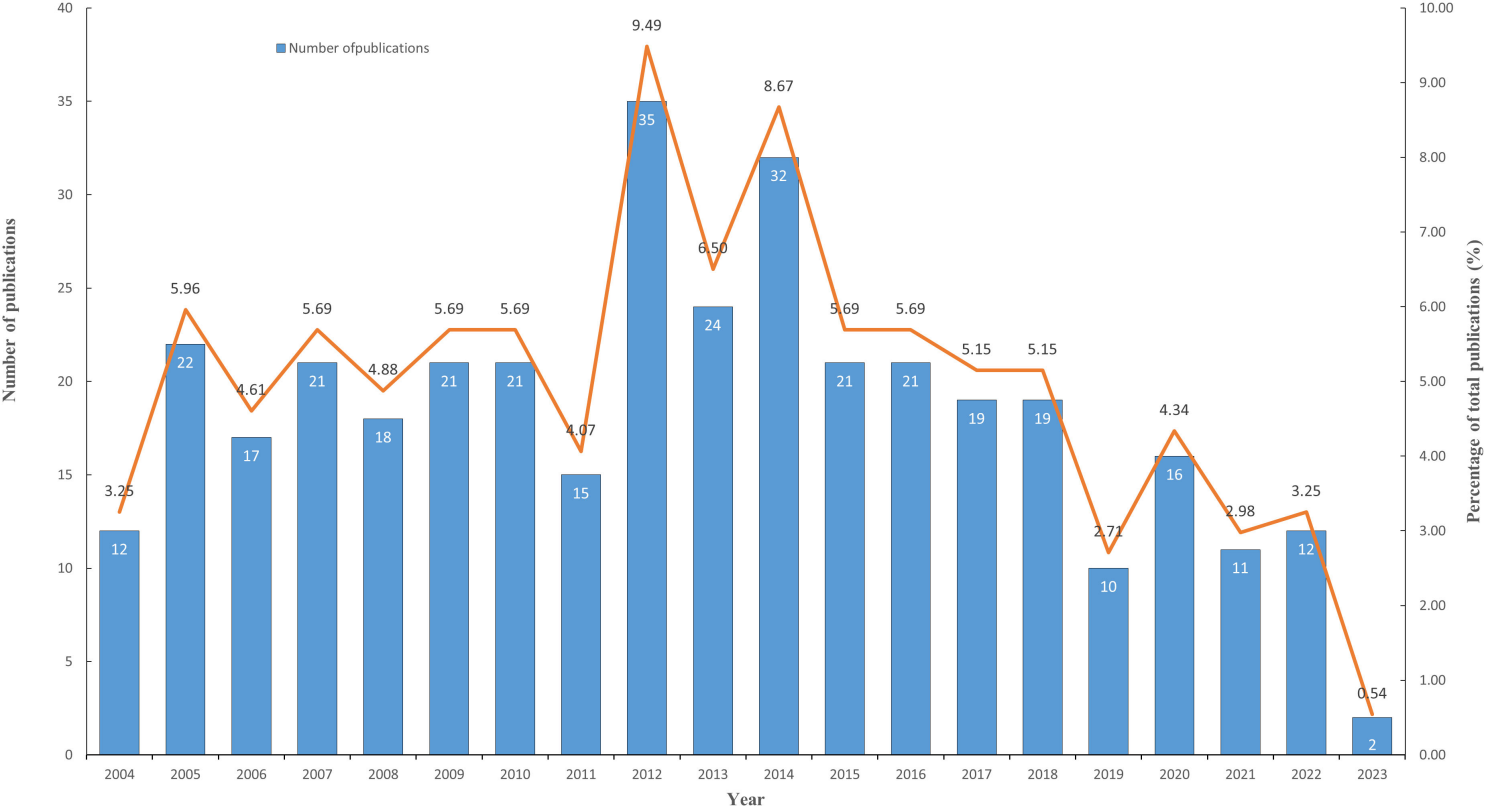
The changing trend of annual publications on breast cancer lymphangiogenesis from 2004 to 2023. (The blue column represents the annual publication number, while the orange line shows the percentage of publications’ development trend.).

This study employed CiteSpace to extract the yearly publication data of the articles included in this study, and Microsoft Excel 2019 for depicting the trend of annual publication. The publication year related to lymphangiogenesis in breast cancer spanned from 2004 until 2023. **Figure 1** illustrates the distribution of publication numbers by year. The annual number of publications in the field was 18. Since 2004, the number of publications in this field has remained fairly consistent, peaking in 2012 (35). The turning point occurred in 2015. Since then, the number of papers published has decreased, potentially signifying that this research field has reached a bottleneck. It is thus critical that prospective research, scientific analyses, and forward direction be addressed promptly.

### 3.2 Analysis of distribution and cooperation of leading countries/regions

**Figure 2:**
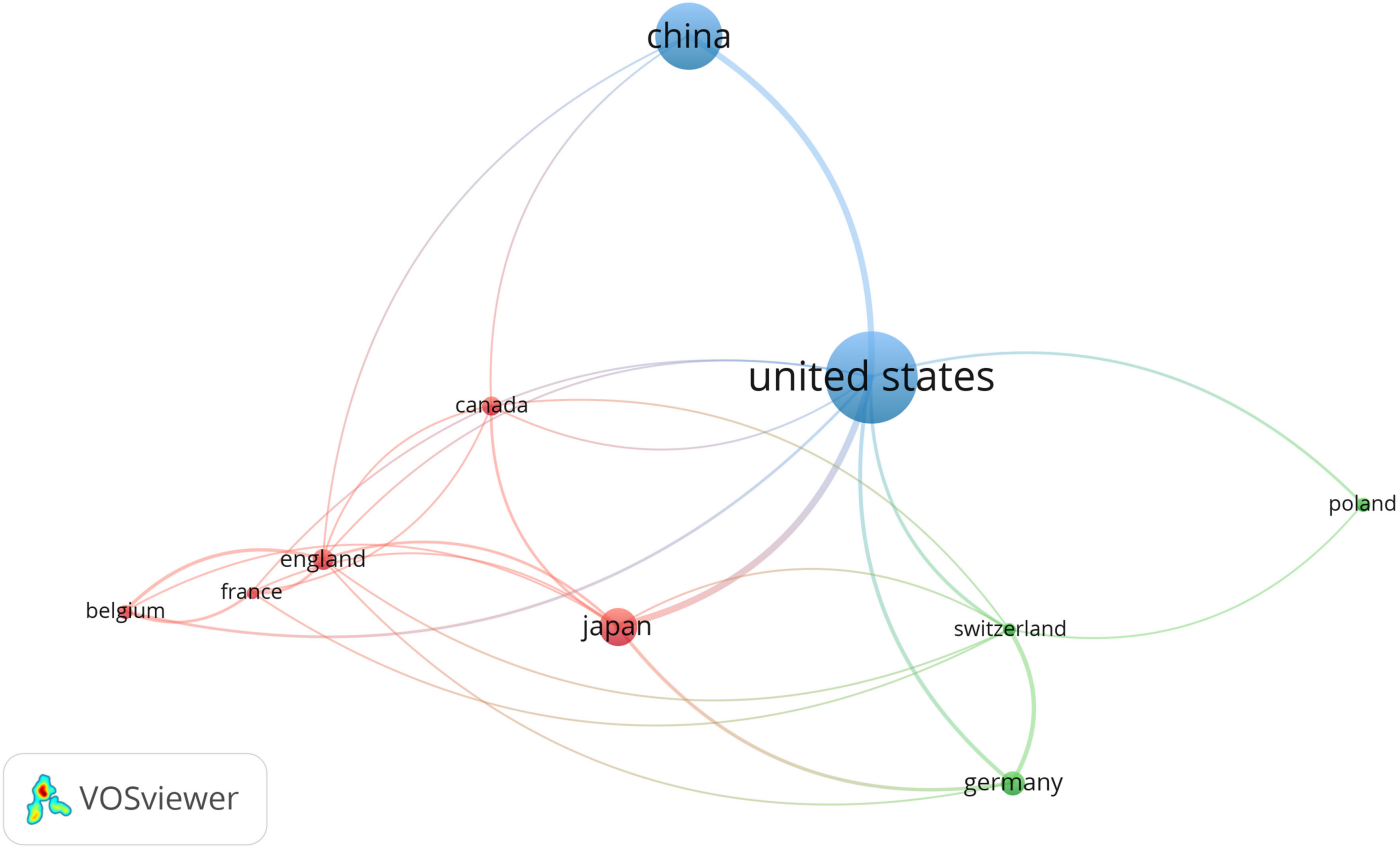
The co-occurrence of the top ten productive countries. (The node size represents the number of articles; the curve thickness indicates the strength of collaboration between countries; and the colors denote various collaboration groups.). From: VOSviewer.

Countries/regions are platforms for researchers to conduct scientific research, providing them with financial, equipment, and human support as well as the rights to the findings. In order to elucidate the extent of involvement of several nations in research regarding lymphangiogenesis in breast cancer, this study carried out a bibliometric analysis of the 42 countries involved. Setting the minimum number of national publications at 14 through VOSviewer, we obtained the top 10 countries in terms of the number of publications (**Table 1**) and visualized the cooperation between them (**Figure 2**). This demonstrates an extremely unbalanced spread of publishing countries in this area and has a significant top effect. The vast majority of the studies have been authored by researchers from a few countries. The US had the largest number of publications (102, 27.64%), followed by China (74, 20.05%) and Japan (43, 11.65%). The top three cited countries were the US, Japan, and China. Additionally, the three countries with the highest average citation rate were the England, Switzerland, and Japan (**Table 1**). The results revealed that the stated countries were more predisposed to researching lymphangiogenesis in breast cancer.

**Table 1:** Top 10 productive countries in the field of lymphangiogenesis in breast cancer.

Rank	Country	Documents	Citations	Average Citation
1	United States	102	5709	55.97
2	China	74	2532	34.22
3	Japan	43	2864	66.60
4	Germany	28	1729	61.75
5	England	24	2163	90.13
6	Canada	22	1003	45.59
7	Belgium	16	1024	64.00
8	Poland	16	493	30.81
9	Switzerland	15	1182	78.80
10	France	14	374	26.71

This study used VOSviewer to analyze the cooperation of different countries. The line between nodes indicates the co-authorship between countries, the larger nodes suggested more postings, and the thicker lines between two nations suggested a stronger degree of cooperation. The findings underlined that the US had more cooperation with China and Japan. The US, with the largest amount of publications, has collaborated with all nations (**Figure 2**). The abovementioned results indicate that the US remains the leader in the research area of lymphangiogenesis in breast cancer while engaging in more interchange and collaboration in the field of lymphangiogenesis in breast cancer.

### 3.3 Analysis of the authors and co-cited authors

The authors’ scientific research activities in a certain subject area and the quantity and quality of their publications are instrumental in advancing the development of the discipline, to some extent. Looking at the works of the most influential authors in the present research field and examining their articles allows us to ascertain the current research trends. According to Price’s Law, the minimum number of articles published by the core authors in a specific field is m =0.749× $\sqrt{n_{max}}$ ≈2.36 $(n_{max}$ is the number of the most productive author’s publication.) Core authors, with 3 or more publications, are hereby perceived as the most prolific authors in this field, resulting in a total of 255 papers written by 65 core authors, amounting to 69.1% (255/369) of the total number of papers. This falls in line with Price’s criterion that half of the papers should be created by a productive set of authors in a certain area. Substituting the above values into the formula computation is also largely consistent with Price’s Law.

It thus appears that a cohesive cohort of researchers has emerged in the field of lymphangiogenesis in breast cancer. Utilizing VOSviewer, we conducted an analysis of 2120 authors and determined the top five authors by setting the minimum number of publications at 7. **Table 2** itemizes the top five with the highest number of publications and citations.

**Table 2:** Top 5 productive authors and co-cited authors in the field of lymphangiogenesis in breast cancer.

Rank	Author	Documents	Citations	Average Citation	Institution	Location	Co-cited author	Citations
1	Mousumi Majumder	10	458	45.80	Brandon University	Canada	Steven A Stacker	238
2	Peeyush K Lala	10	444	44.40	University of Western Ontario	Canada	Sebastian F Schoppmann	200
3	Kazuaki Takabe	9	565	62.78	Virginia Commonwealth University School of Medicine and the Massey Cancer Center	United States	Mihaela Skobe	196
4	Marc G Achen	7	731	104.43	University of Melbourne	Australia	Satoshi Hirakawa	162
5	Michael Detmar	7	477	68.14	Swiss Federal Institute of Technology	Switzerland	Marc G Achen	148

Mousumi Majumder and Peeyush Lala, the two most productive authors, collaborated on nine articles related to lymphangiogenesis in breast cancer (19–27). Following an assessment of the published studies, the two scientists persisted in their research on this topic from 2012 to 2021, with a focus on prostaglandins (PGs) and their role in this condition. They found that PG, particularly Prostaglandin E2 (PGE2), significantly influences breast cancer-associated lymphangiogenesis. Moreover, Cyclooxygenase (COX)-2 expression by breast cancer cells or tumor stroma leads to high PGE2 levels in the tumor milieu. PGE2 and prostaglandin E2 receptor EP4 (EP4) subtype combine on multiple cell types: tumor cells, tumor-infiltrating immune cells, and lymphatic endothelial cells (LECs), EP4 activation on cancer cells, and macrophages upregulated vascular endothelial growth factor c/d (VEGF-C/D) production. Their purpose is to stimulate the sprouting of LECs and promote lymphangiogenesis and lymphatic metastases. The ligation of EP4 with PGE2 on cancer or host cells can initiate a new cascade of molecular events resulting in the cross-talk between cancer cells and LECs, facilitating lymphangiogenesis and the lympho-vascular transport of cancer cells. Hence, EP4 can be considered a promising therapeutic target for lymphangiogenesis in breast cancer (19, 21, 22, 24, 26).

### 3.4 Analysis of leading journals

According to Bradford’s law, this investigation comprised a total of 199 journals, segmented into three sections based on the number of publications. Moreover, the number of publications in each area was generally the same, and the ratio of journal numbers was close to 1:3:9 (1:3:3^2^). The journal distribution of studies in the realm of lymphangiogenesis conforms to Bradford’s law. **Table 3** lists the three areas of 199 journals based on the number of publications. Finally, 17 journals were considered as core journals in this field. **Table 4** displays the 17 core journals and their IF, JCR, and H-index.

**Table 3:** The three areas of 199 journals based on the number of publications. Area.

Area	Publications/Journal	No. of Journal	No. of Publication
Core Area	≥5	17	117
Related Area	2-4	49	119
Non-related Area	1	133	133

**Table 4:** The Top 17 productive journals associated with lymphangiogenesis in breast cancer.

Rank	Journal	Country	Documents	Citations	Average Citation	IF (2022)	JCR (2022)	H-index
1	Cancer Research	United States	13	1129	86.85	11.2	Q1	466
2	British Journal Of Cancer	England	10	961	96.10	8.8	Q1	248
3	Clinical Cancer Research	United States	9	1139	126.56	11.5	Q1	344
4	Anticancer Research	Greece	8	146	18.25	2.0	Q4	126
5	BMC Cancer	England	8	271	33.88	3.8	Q2	139
6	PLoS One	United States	8	329	41.13	3.7	Q2	404
7	Breast Cancer Research	England	6	296	49.33	7.4	Q1	158
8	Cancer Science	United States	6	224	37.33	5.7	Q2	150
9	Cancers	Switzerland	6	88	14.67	5.2	Q1	92
10	Clinical & Experimental Metastasis	Netherlands	6	215	35.83	4.0	Q2	103
11	International Journal Of Molecular Sciences	Switzerland	6	218	36.33	5.6	Q1	195
12	Oncology Reports	Greece	6	114	19.00	4.2	Q3	108
13	American Journal of Pathology	United States	5	320	64.00	6.0	Q1	297
14	Breast Cancer Research and Treatment	United States	5	331	66.20	3.8	Q2	171
15	Faseb Journal	United States	5	172	34.40	4.8	Q1	297
16	International Journal of Cancer	Switzerland	5	427	85.40	6.4	Q1	251
17	Oncogene	England	5	529	105.80	8.0	Q1	360

Cancer Research, which had an IF of 11.2 in 2022, produced the greatest volume of research in the cancer field, with the British Journal of Cancer (BJC) and Clinical Cancer Research (CCR) in second and third place, respectively. Equally important, CCR had the highest impact factors. From an annual IF standpoint, CCR, Cancer Research, and BJC are viewed as authoritative journals in this field. The JCR standard of 2022 demonstrates that 10 out of the 17 most productive journals fall into the Q1 category, with the remaining 5 in Q2, testifying to the high quality of research in this field.

### 3.5 Analysis of keywords

**Figure 3:**
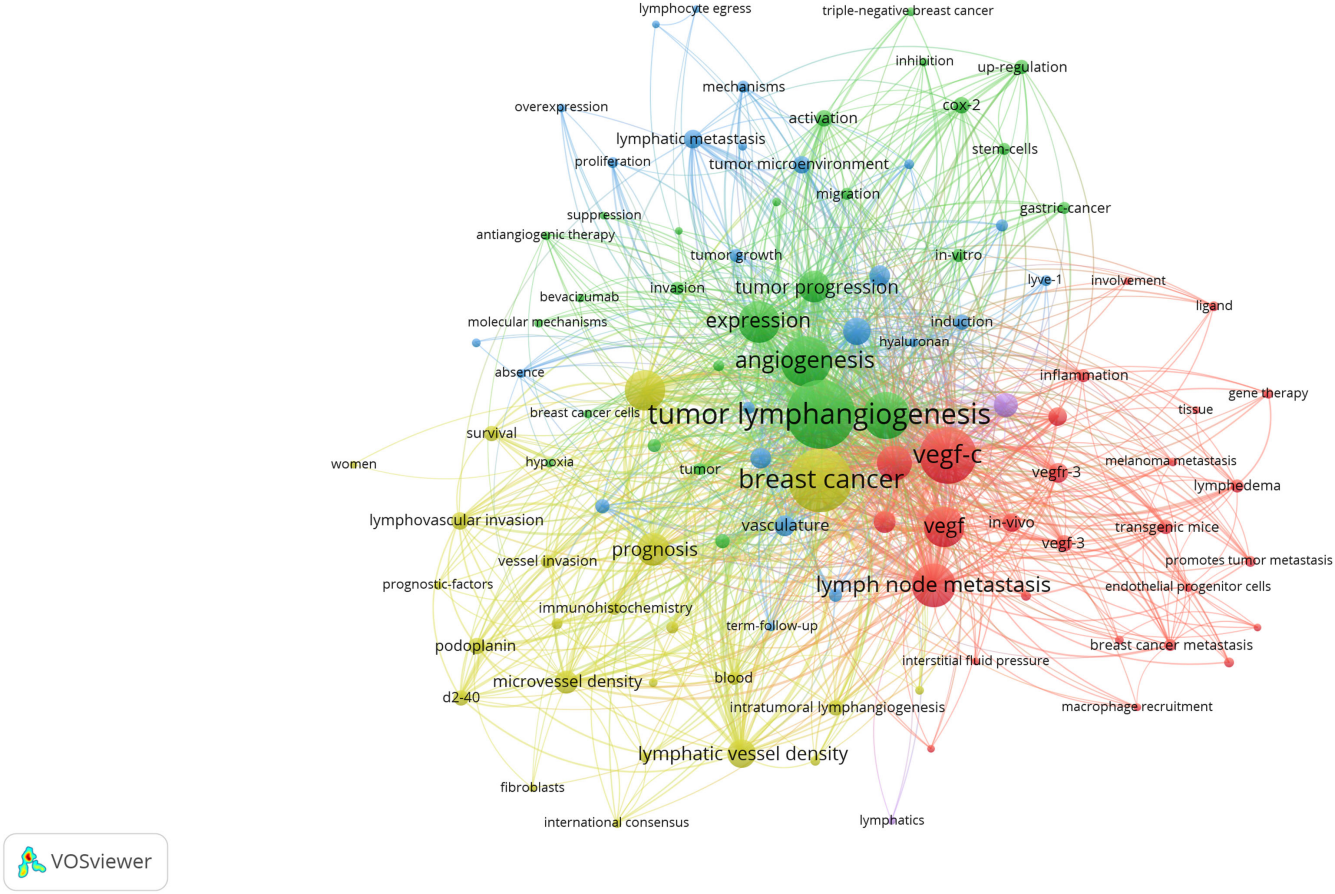
Network visualization of keywords drawn by VOSviewer. [The size of the nodes represents the number of occurrences; the thickness of the curve represents the strength of connection; the different colors represent the different clusters. The key words were classified into 4 clusters, presented by four colors (red, green, blue, and yellow)]. From: VOSviewer.

**Figure 4:**
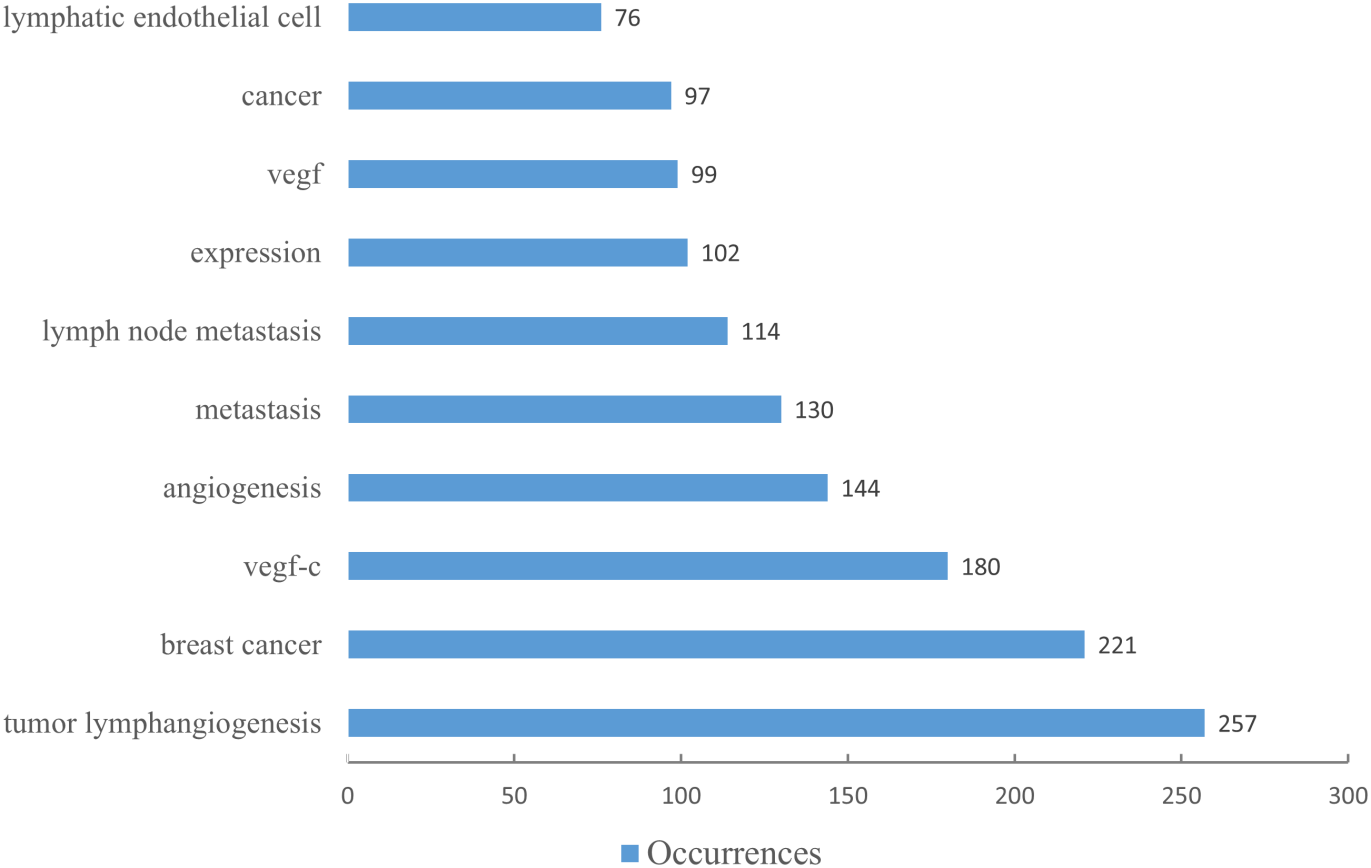
The top ten keywords by number of occurrences. (The blue column indicates the number of occurrences.).

Utilizing keywords as signifiers of an article, an examination of said keywords can enable us to rapidly comprehend the theme of the article and grasp the research hotspots and research frontiers in this domain. A rise in the prevalence of keyword citations or a greater frequency of keyword occurrence over a certain interval serves as an indicator for evaluating the most cutting-edge topics or emerging trends. Therefore, the keyword analysis of references in a certain field can reveal current research hotspots and predict the direction of future research hotspots through the frequency of keyword occurrences. There are a total of 1407 keywords appearing in 369 articles pertaining to lymphangiogenesis in breast cancer. VOSviewer was used to draw a keywords co-occurrence diagram in this field, and keywords with a frequency greater than 5 were selected for visualization (**Figure 3**). When the threshold was selected at the value of “4,” 137 keywords met the conditions, and the visualization map was somewhat mixed. Meanwhile, when the threshold was set to “6,” 90 keywords conformed to the criterion, and the map was somewhat straightforward. Against this backdrop, we used the threshold value of 5. At this time, there were 103 keywords to meet, the conclusion was evident, and the distinct branches of the field could be observed. **Figure 4** shows the top 10 keywords in terms of their frequencies.

**Figure 5:**
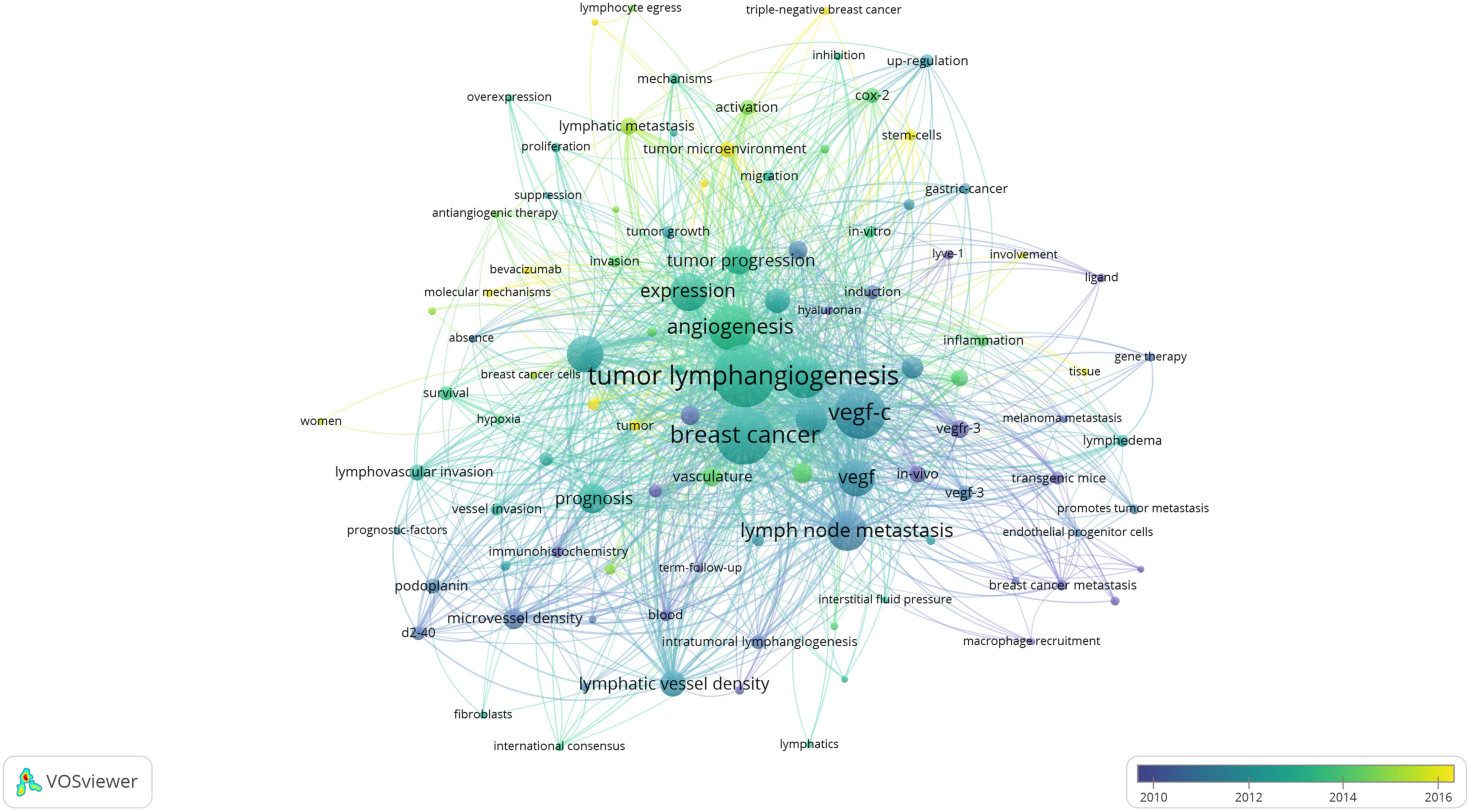
An overlay visualization of keywords drawn using VOSviewer. (The node size represents the number of occurrences; the curve thickness indicates the connection strength; and the colors denote the average publication years, with keywords in blue appearing before those in yellow.). From: VOSviewer.

**Figure 3** illustrates that all keywords revolve around the topic of “tumor lymphangiogenesis” and are divided into four main clusters: I. keywords associated with the prognosis and related detection indicators of tumor lymphangiogenesis (yellow clusters), II. keywords centered around the vascular endothelial growth factor (VEGF) (red and purple clusters), III. keywords linked to the influence of tumor lymphangiogenesis and angiogenesis on tumor progression (green clusters), and IV. keywords related to the metastasis mechanism of the lymphatic system and the role of tumor microenvironment (blue clusters). **Figure 5** displays the overlay visualization map of the 103 keywords. The evolution of high-frequency keywords over time can provide an intuitive insight into the research focus. The yellow nodes were indicative of the keywords that were emerging around 2016. Among these, tumor microenvironment (TME), epithelial-mesenchymal transition (EMT), stem cells, triple-negative breast cancer, and bevacizumab may become the future research hotspots in the field of lymphangiogenesis in breast cancer.

### 3.6 Analysis of burst keywords

**Figure 6:**
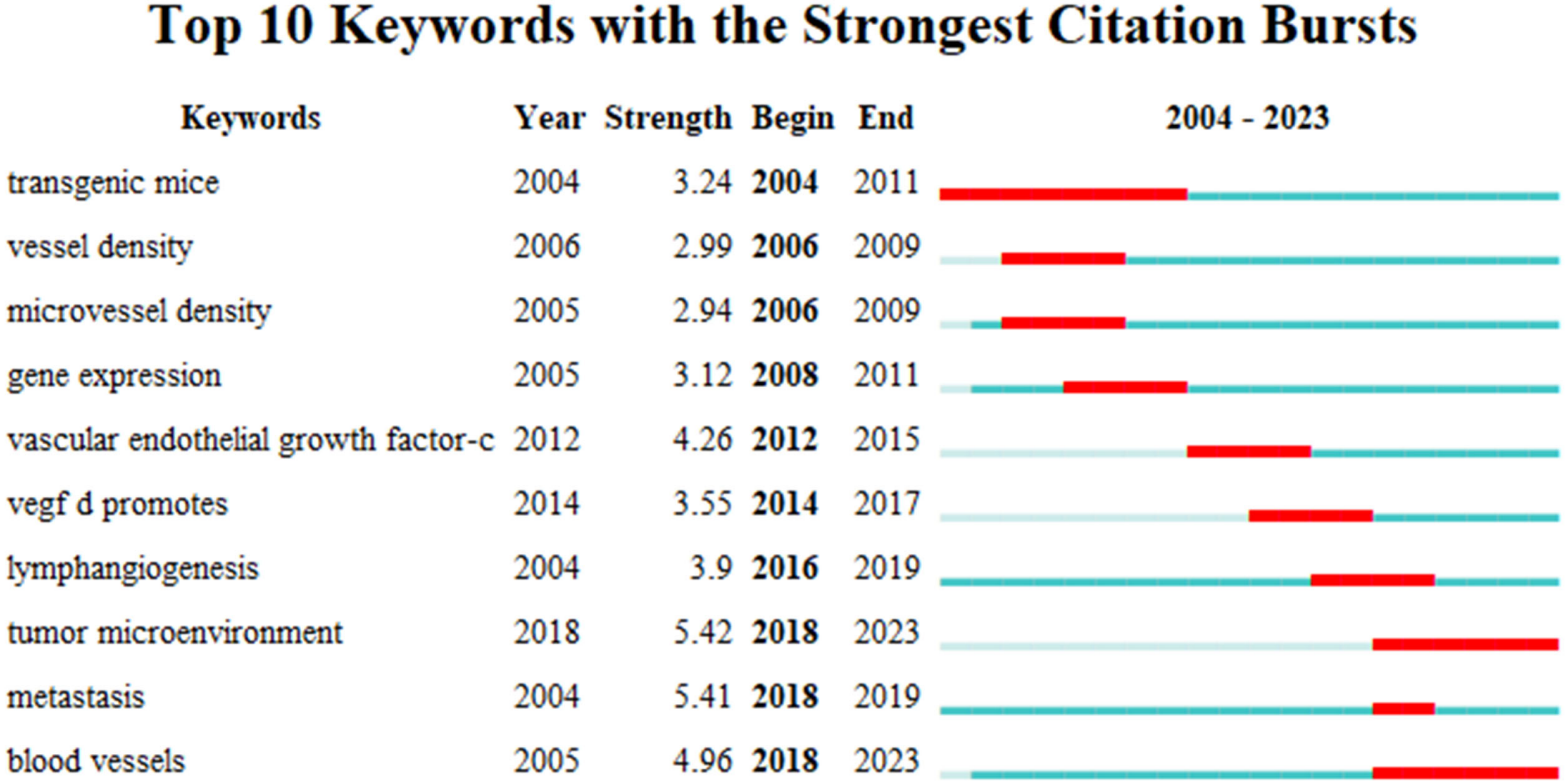
The top 10 burst words. (The strongest citation burst indicates a significant change in a variable over a short period, with red bars signifying the burst duration.).

Burst words represent new discoveries or turning points in the development of a research field. They have been subject to extensive research in a short time, represent the frontier of the field, and can highlight the hotspots of future research and the evolution of the discipline. Applying CiteSpace, the research hotspots and emerging trends of lymphangiogenesis in breast cancer over a period of time were identified and analyzed by recognizing and studying keywords with citation bursts. The map presents the top 10 keywords with the strongest citation bursts from 2004 to 2023 (**Figure 6**). The red bar shows that the keyword was frequently cited, and the green bar indicates that the keyword was rarely cited. In the early days (2004-2011), “transgenic mice,” “vessel density,” “microvessel density,” and “gene expression” appeared. Meanwhile, in the mid-term (2012–2017), “vascular endothelial growth factor-c,” “lymphangiogenesis,” and “vegf-d promotes” emerged, and in the later stage (from 2018 to the present), “tumor microenvironment,” “metastasis,” and “blood vessels” surfaced. Among them, “transgenic mice” had a burst strength of 3.24, implying that it garnered the most attention and research from 2004 to 2011. Then, the keyword “tumor microenvironment” received the strongest citation burst (strength = 5.42) from 2018 to 2023, followed by “metastasis” (strength = 5.41), and “blood vessels” (strength = 4.96), which highlights that these three could represent important content and frontiers in the research domain of lymphangiogenesis in breast cancer and could remain a hot research topic today and in the coming years.

### 3.7 Analysis of references co-citation

**Figure 7:**
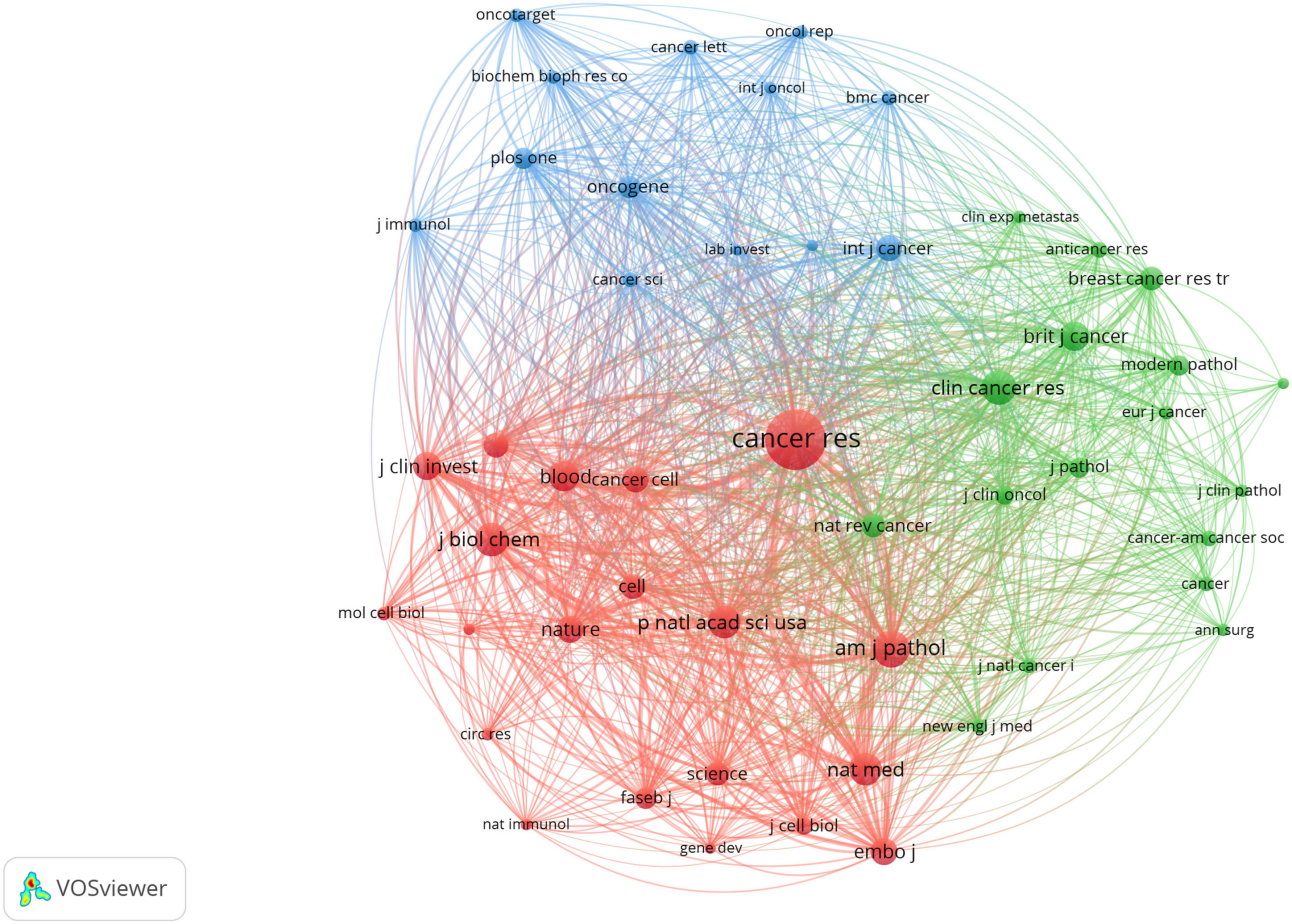
The co-occurrence of the top 50 cited journals. (The node size represents the number of articles; the curve thickness indicates the connection strength between journals; and the colors denote varying journal types.).

VOSviewer was employed to filter the top 10 cited articles among 369 articles, and the minimum number of citations was set to 261 to generate **Table 5**. Among the 10 cited articles, 6 were review articles, primarily focusing on the role and molecular mechanisms of lymphangiogenesis in cancer progression. Applying VOSviewer to filter 12458 citations from 1801 journals and establish the minimum number of citations to 85, we identified the top 50 cited journals. **Figure 7** shows their co-citation relationships. A profound nexus between journals concerning lymphangiogenesis in breast cancer was unveiled, the three highest-cited journals being Cancer Research, American Journal of Pathology, and Clinical Cancer Research. **Table 6** details the top 10 co-cited journals ranked by the number of citations. The co-citation network of the journal comprised three clusters, including journals related to medical oncology, biochemistry, and molecular biology (red cluster), tumor-pathology-related (green cluster), and cancer direction (blue clusters).

**Table 5:** Top 10 cited papers in the field of lymphangiogenesis in breast cancer.

Rank	Title	Author	Citations	Journal	Year	Type
1	Lymphangiogenesis: Molecular mechanisms and future promise	Tuomas Tammela	975	Cell	2010	Review
2	Lymphangiogenesis and lymphatic vessel remodelling in cancer.	Steven A Stacker	501	Nature Reviews Cancer	2014	Review
3	PDGF-BB induces intratumoral lymphangiogenesis and promotes lymphatic metastasis.	Renhai Cao	393	Cancer Cell	2004	Article
4	Role of tumor associated macrophages in tumor angiogenesis and lymphangiogenesis.	Vladimir Riabov	386	Frontiers In Physiology	2014	Review
5	Multi-kinase inhibitor E7080 suppresses lymph node and lung metastases of human mammary breast tumor MDA-MB-231 via inhibition of vascular endothelial growth factor-receptor (VEGF-R) 2 and VEGF-R3 kinase.	Junji Matsui	375	Clinical Cancer Research	2008	Article
6	Lymphatic vasculature: development, molecular regulation and role in tumor metastasis and inflammation.	Pipsa Saharinen	324	Trends in Immunology	2004	Review
7	The Contribution of Angiogenesis to the Process of Metastasis.	Diane R Bielenberg	288	Cancer Journal	2015	Review
8	M-CSF inhibition selectively targets pathological angiogenesis and lymphangiogenesis.	Yoshiaki Kubota	288	Journal of Experimental Medicine	2009	Article
9	The VEGF-C/Flt-4 axis promotes invasion and metastasis of cancer cells.	Jenliang Su	279	Cancer Cell	2006	Article
10	Molecular biology and pathology of lymphangiogenesis.	Terhi Karpanen	269	Annual Review of Pathology	2008	Review

**Table 6:** Top 10 co-cited journals ranked by the number of citations.

Rank	Journal	Citations	IF 2022	JCR 2022	Country	H-index
1	Cancer Research	1428	11.2	Q1	United States	466
2	American Journal of Pathology	631	6.0	Q1	United States	297
3	Clinical Cancer Research	562	11.5	Q1	United States	344
4	Journal Of Biological Chemistry	549	4.8	Q2	United States	544
5	Proceedings Of The National Academy Of Sciences Of The United States Of America	517	11.1	Q1	United States	838
6	Nature Medicine	498	82.9	Q1	United States	605
7	Blood	440	20.3	Q1	United States	506
8	British Journal Of Cancer	432	8.8	Q1	England	248
9	Journal Of Clinical Investigation	395	15.9	Q1	United States	527
10	Embo Journal	383	11.4	Q1	United States	417

## 4 Discussion

Through the application of VOSviewer and CiteSpace software, this study conducted a bibliometric analysis of the literature surrounding lymphangiogenesis in breast cancer. This enabled researchers to swiftly and methodically acquire the development trajectory, current research status, and focal points in the area of lymphangiogenesis in breast cancer from 2004 to 2023, thereby predicting the global trends in this field. From 2004 to 2023, a total of 369 articles on lymphangiogenesis in breast cancer were published. Between 2004 and 2012, there was a steady rise in the number of publications, and from 2015 to 2023, the figure decreased, likely signifying a plateau in this area of research. This bottleneck could be due to the following reasons. The 2013 St. Gallen International Breast Cancer Consensus Conference redefined breast cancer molecular typing, dividing breast cancer into four subtypes: luminal A, luminal B, HER2-positive, and basal-like or triple-negative (TNBC) phenotype (28, 29). It marks the beginning of the precision treatment era for breast cancer based on molecular typing, as well as the emergence of numerous low-toxic and efficient molecularly targeted drugs for various molecular types. For example, the Food and Drug Administration (FDA) approved patozumab and TDM-1 in HER2-positive advanced breast cancer patients in 2012 and 2013, respectively (30, 31), Pfizer’s Palbiclib as an initial endocrine therapy scheme in 2015, and the PARP inhibitor Olaparib for treating BRCA1 or BRCA2 gene mutation breast cancer patients (32). Marketing these drugs has significantly benefited breast cancer patients. Apart from the research and development of new drugs and current treatment schemes with varying action mechanisms, including combining chemotherapy with targeted therapy, immunotherapy, and biological therapy, it offers a novel approach to breast cancer. Therefore, researchers shifted their focus away from traditional anti-tumor angiogenesis and anti-tumor lymphangiogenesis and toward investigating the biological behavior and therapeutic targets of various breast cancer molecular subtypes, thereby reducing the research literature on breast cancer lymphangiogenesis. In this context, prospective research, scientific analysis, and forward-looking guidance are imperative.

The research development in various countries or regions in the field of lymphangiogenesis in breast cancer is unbalanced. The majority of these articles originate from US, China, and Japan-based institutions and researchers, with the US having the most consistent collaborations with China and Japan. It is worth noting that the country with the highest citations is the US, while the UK has the highest average citation. However, the average citation rate in China stands at only 34.22, and there are no authors from Chinese institutions represented in the set of exceptionally productive authors who have published more than 7 articles. This indicates that the Chinese research community benefits from a larger population and the ability to produce more research and literature, though the quality of the studies still needs to be improved. Ultimately, Western researchers have a greater bearing on the topic of lymphangiogenesis in breast cancer. From the perspective of journals, it is found that journals in the core area are consistent with Bradford’s Law in terms of journal distribution. Among the 17 core journals, 10 belong to Q1, and 9 of the top 10 co-cited journals belong to Q1, emphasizing that articles associated with lymphangiogenesis in breast cancer are of high quality and have high scientific reference value.

In the keyword analysis, the keywords are divided into four clusters. The yellow clusters stand for the detection indicators and prognosis connected to lymphangiogenesis in breast cancer. Among them, the high frequency of “lymphatic vessel density,” “microvessel density,” “d2-40,” “diagnosis,” “lymphatic invasion,” and “survival” suggests that these keywords have garnered considerable interest. Lymphatic vessel density (LVD) or microvessel density (MVD) is a surrogate marker of tumor lymphangiogenesis. In view of this, quantitative assessment of lymphangiogenesis or angiogenesis in breast cancer can more precisely determine those with high recurrence risk, particularly those with negative lymph nodes, which is of value in the prognosis and treatment of breast cancer at its early stages. High LVD has adverse effects on disease-free survival (DFS) (HR 2.02, 95% CI 1.69 to 2.40) and OS (HR 2.88, 95% CI 2.07 to 4.01). LVD has a greater prognostic significance than vascular density in breast cancer. High LVD and the presence of lymphovascular invasion both predict poor prognosis in breast cancer (33).

Immunohistochemistry was put forward by Van der Auwera et al. as the method to detect LVD (34), however, there is a lack of clinical consensus on the quantitative method for LVD. Weidner’s initial strategy from 1991 is regularly utilized to assess LVD (35), a widely adopted method, to quantify intratumoral angiogenesis in breast cancer. Weidner’s approach commences with the identification by light microscopy of the area of highest neovessel density (the “hotspot”), by scanning the entire tumoral section at low power. Then, individual microvessels are counted at a higher power (×200 field) in a suitable area. However, there are several issues. Firstly, the amplification factor chosen for representative regions in different studies can vary, leading to discrepancies in the number of lymphatic vessels present in the region and resulting in distinct results concerning the number of lymphatic vessels tallied. Secondly, since the asset value of lymphatic vessel density is not a normal distribution, its data representation varies, yielding a different cutoff value. Finally, there are no established standards for the classification of high, medium, and low lymphatic vessel densities. Therefore, studies with more standardized and stringent designs are required in the assessment of LVD, especially in the methods of counting the number of lymphatic vessels, the selection of appropriate critical values, and the gradient classification of lymphatic vessel density, which are crucial points to consider.

The lymphatic endothelial-specific markers used for detecting lymphangiogenesis primarily include vascular endothelial growth factor receptor 3 (VEGFR-3), prospero homeobox protein 1 (Prox-1), lymphatic endothelial receptor-1 (LYVE-1), platelet endothelial cell adhesion molecule-1 (CD31), podoplanin, and D2-40 (36). Our research findings underscore that podoplanin and D2-40 had a higher frequency in the keyword clusters network, suggesting that these two markers hold a significant role in lymphangiogenesis. Podoplanin, a transmembrane protein, is highly expressed on the surface of LECs but not on vascular endothelial cells, thus rendering it a distinctive marker for LECs (37). The widely used commercial monoclonal antibody D2-40 binds to a fixation-resistant epitope of podoplanin (38). Additionally, in comparison to Prox-1 and LYVE-1, it is more sensitive in recognizing lymphangiogenesis in breast cancer and displays the strongest immunoreactivity in both intratumoral and peritumoral LECs (39–41). The detection of these distinct lymphatic endothelial markers renders us able to differentiate between lymphatic and blood vessels with efficacy (42). With the development of immunohistochemistry technology, we can utilize specific markers like podoplanin/D2-40/LYVE-1/Proxl to evaluate lymphangiogenesis in our research (43, 44).

VEGF-C and VEGF-D, two components of the vascular endothelial growth factors (VEGFs) family, are the first and sole representatives of vascular endothelial growth factors that possess specificity for lymphatic endothelial cells (45, 46). Simultaneously, burst words showed that from 2012 to 2017, the strength of VEGF-C and VEGF-D were 4.26 and 3.55, respectively, demonstrating that VEGFs linked to lymphangiogenesis received more attention. During this period, investigations into the molecular mechanisms of lymphangiogenesis continued. The overexpression of VEGF-C and VEGF-D can stimulate lymphangiogenesis and is associated with lymphovascular invasion and lymph node metastasis (47–49). VEGFR-3, as a receptor for VEGF-C and VEGF-D, is overexpressed and solely expressed in LECs. Meanwhile, VEGF-C and VEGF-D can activate VEGFR-3, enabling LECs to bud from embryonic veins and initiating the formation of primitive lymphatic sacs, thereby playing a vital role in the earliest stages of lymphatic vessel development (45). On the other hand, the interaction between VEGF-C/VEGF-D and VEGFR-3 triggers receptor phosphorylation and activates the Akt signaling pathway, which ultimately promotes the survival, proliferation, and migration of LECs, subsequently leading to the lymphangiogenesis in breast cancer (50). The intra- and peri-tumoral LVD of invasive breast cancer was quantified through the D2-40 immunohistochemical method. Studies showed that the high expression of VEGF-C/D in breast cancer may induce lymphangiogenesis in the peri-tumoral region, leading to elevated levels of peri-tumoral LVD, increased tumor invasiveness, and a higher risk of lymph node invasion, distant metastasis, and a poor prognosis (49, 51).

The green clusters demonstrate the effect of tumor lymphatic vessels and angiogenesis on tumor progression. The lymph nodes in the drainage area of the breast are the most common sites for metastasis of breast cancer cells (52). Cancer cells must undergo lymphangiogenesis in order for them to metastasize through the lymphatic vessels. The newly formed lymphatic vessels provide pathways for cancer cells to reach regional lymph nodes, thus priming cancer cells for distant metastasis (51). Meanwhile, lymphangiogenesis also expands the contact area between invasive cancer cells and LECs, contributing to metastasis (53). Therefore, lymphangiogenesis is the pivotal element to stimulate the invasion and metastasis of breast cancer.

The network visualization of keywords has revealed that in recent times, there has been an increased focus on the TME, epithelial-mesenchymal transformation (EMT), stem cells, triple-negative breast cancer, and bevacizumab in the field of lymphangiogenesis. Simultaneously, the analysis of burst words has unveiled that “tumor microenvironment” has been gaining traction in the research field since 2018. Therefore, we anticipate TME to become a focus of research into lymphangiogenesis with regard to breast cancer going forward. Growing evidence shows that the progression of a tumor to a malignant phenotype is heavily dependent on the interplay between tumor cells and the TME. Different TME components may stimulate or inhibit tumor progression by regulating gene expression in tumor cells and stromal cells. Lymphangiogenesis is generated by the dynamic interaction of breast cancer cells, lymphatic endothelial cells, and TME (54, 55).

VEGF-C/D, secreted by tumor cells, functions upon VEGFR-3, providing a basic signal for the proliferation, migration, and tubular structure formation of LECs (56). Along with passively receiving signals from tumor cells, LECs can also engage with multiple cytokines, such as chemokines, extracellular matrix (ECM), immune cells, tumor-associated macrophages (TAM), inflammatory cytokines, and other cytokines, thereby fostering lymphangiogenesis and tumor progression. These cytokines are important TME components. Chemokines, a class of small cytokines secreted by tumor cells, are essential in stimulating the migration of LECs and prompting tumor-related lymphangiogenesis when combined with their receptors. Among all known chemokine receptors, chemokine receptor 4 (CXCR4) and chemokine receptor 7 (CCR7) display a relatively high expression in breast malignant tumors and metastatic tissues (57). Moreover, CXCR4 and CCR7 interact with their respective ligands chemokine (C-X-C motif) ligand 12 (CXCL12) and chemokine (C-X-C motif) ligand 21 (CCL21), enabling tumor cells to have “chemotaxis” and promoting their metastasis to the intended organs (23, 58).

The relationship between LECs and ECM is fundamental to tumor lymphangiogenesis (59). On the one hand, ECM proteins, consisting of collagen, laminin, and fibronectin, can facilitate the proliferation of LECs and the development of tumor lymphangiogenesis by providing essential structural backing (60). On the other hand, ECM remodeling, catalyzed by matrix metalloproteinases (MMPs), may generate a more conducive environment for LECs, thereby promoting lymphangiogenesis (61). MMP-2, as an efficient collagenase, can effectively drive LECs to migrate to distant areas through the collagen matrix. Invasive cancer cells actively recruit stromal cells and interact with them to create a TME suitable for tumor growth and potential metastasis. Meanwhile, TME remodeling results in excessive amounts of low molecular weight hyaluronic acid (HA) in the TME, further activating macrophages to demonstrate lymphatic endothelial characteristics and encourage tumor lymphangiogenesis (62). This finding reveals a new mechanism of lymphangiogenesis induced by TME remodeling and provides a potential target for inhibiting the lymphatic metastasis of breast cancer.

Recent evidence shed light on the bidirectional crosstalk between LECs and immune cells. Through the action of immune cells, white blood cells are mobilized to produce VEGF-C and secrete some lymphangiogenic factors, such as lipocalin 2 (LCN2) from macrophages. This activates VEGF-C through a multicellular signaling pathway, thus promoting lymphangiogenesis in the TME (62). Additionally, cytokines produced by infiltrating immune cells can directly affect the proliferation, migration, and tubular structure formation of LECs. For instance, IL-17 secreted from Th17 cells can induce LEC proliferation, hence promoting lymphangiogenesis (63, 64).

Concerning the occurrence and development of lymphangiogenesis, tumor-associated macrophages (TAM) are also involved. TAM expresses a considerable amount of (a) VEGF-C/D, which promotes lymphangiogenesis, and (b) lymphatic endothelial progenitor cells that can be transformed into LECs under pathological conditions, serving as a direct structural contributor to new lymphatic vessels (65). Weichand et al. observed that the sphingolipid sphingosine-1-phosphate (S1PR1) signaling circuit in TAM facilitated lymphangiogenesis via the inflammasome component Nlrp3-dependent IL-1β secretion (66). In addition to the aforementioned TME factors, inflammation-induced lymphangiogenesis should not be overlooked, encompassing IL-1 β recruiting M2 type macrophages that can promote tumor lymphangiogenesis and lymph node metastasis (67). The anti-cytokine IL-10 with anti-inflammatory properties can upregulate the expression of VEGF-C in macrophages, thereby augmenting lymphangiogenesis (68). Among them, Prox1 and NF-kappaB co-regulate LECs, causing an increased expression of VEGFR-3 on the surface of LECs, thus disclosing the mechanism of inflammation-induced lymphangiogenesis (69).

Among the top 10 cited articles pertaining to the lymphangiogenesis of breast cancer, 6 were focused on the review of the essential concepts. All six articles furnish a review of the molecular mechanisms of lymphangiogenesis. Collectively, through these articles, we could grasp the structure of lymphatic vessels, the development and remodeling of tumor lymphangiogenesis, the function of lymphatic vessel growth factors in lymphangiogenesis, and the influence of lymphangiogenesis on tumor metastasis. The other four articles are specialized. Among them, the platelet-derived growth factor BBs (PDGF-BB) are novel potent and direct lymphangiogenic factors that can induce tumor lymphangiogenesis and promote lymphatic metastasis. Additionally, the blockage of the VEGF-C/-D-VEGFR-3 axle does not block PDGF-BB-induced lymphangiogenesis *in vitro* and *in vivo*. It is imperative in stimulating tumor lymphangiogenesis and lymphatic metastasis. Therefore, the formulation of antagonists for PDGF-BB may be a beneficial method for the control of tumor growth and metastasis (70).

Investigations into the VEGF-C/Flt-4 axis have revealed its capacity to take part in tumor progression by promoting the movement and invasiveness of epithelial tumor cells, as well as LEC proliferation (e.g., lymphangiogenesis) and possible vascular endothelial cell proliferation and migration (e.g., angiogenesis). With regard to controlling tumor growth or metastasis, targeting the VEGF-C/Flt-4 axis may be an effective method (71). Concerning the inhibition of tumor lymphangiogenesis and metastasis, multi-kinase inhibitor E7080 and macrophage colony-stimulating factor (M-CSF) have produced newly identified results. E7080 functions as a dual tyrosine kinase inhibitor for VEGFR-2 and VEGFR-3. It has been demonstrated to effectively inhibit both MVD and LVD in the lymph node metastasis of a transplanted tumor in a nude mouse model of breast cancer, showing greater efficacy in anti-lymphangiogenesis and anti-angiogenesis than bevacizumab, and can effectively impede regional lymph nodes and distant lung metastasis (72). M-CSF, a cytokine required for monocyte lineage cell differentiation, can selectively inhibit tumor angiogenesis and lymphangiogenesis while reducing tumor recurrence after treatment interruption (73). It is evident from these results that E7080 and M-CSF are ideal strategies for inhibiting lymphangiogenesis and progression in breast cancer. These extensively referenced texts offer insight into the clinical and basic research of lymphangiogenesis in breast cancer, aiding in the comprehension of the pathological mechanism of tumor lymphangiogenesis and forming a platform from which to explore new mechanisms.

Although lymphangiogenesis has been proposed to be a potential target for preventing cancer metastasis, there are few antilymphangiogenic drugs approved for clinical trials. The main obstacles are the heterogeneity involved in tumor-induced lymphangiogenesis in gene signature, structure, cellular origins and functional plasticity, which is governed by complex factors (74). Elucidating the mechanism that causes lymphangiogenesis heterogeneity in solid tumors may provide better insight into the role of lymphangiogenesis in tumor progression and potential implications for tumor treatment. In addition, similar to tumor angiogenesis, multiple lymphangiogenic pathways are active in some tumor microenvironments, meaning that single pathway targeting drugs might not be efficient in all cases.

## 5 Strengths and limitations

To the best of our knowledge, this is the first scientometrics-based bibliometric examination of the development and trends of lymphangiogenesis in breast cancer. However, several potential limitations exist in our study. First, this research was restricted to certain studies (the documents in SCI-E of WoSCC, English language, publication type of article, and review) in order to meet the format requirements of CiteSpace and VOSviewer. Inevitably, this led to the partial inclusion of articles, thus this study may not fully represent the available information in this domain. Given that a bibliometric analysis does not address a specific research query, narrow areas of the topic were not identified. Secondly, due to the regular updating of the database, there was a certain lag in the data obtained, such as the number of articles, citations, and H-index. Finally, evaluating prior literature, the dearth of keywords or abstracts may increase the chances of being excluded due to poor discoverability.

Notwithstanding the limitations, bibliometric analysis is advantageous for gaining keener insight into the essential components and a far-reaching quantity of studies on lymphangiogenesis in the context of breast cancer. Our study clearly displayed the countries/regions, institutions, authors, journals, references, keywords, and other elements of literature related to lymphangiogenesis in breast cancer in the past 20 years. We collated data sets and reviewed the publications of experts in relevant fields, which enabled us to understand the development process, research status, and knowledge hotspots of lymphangiogenesis in breast cancer. Through keyword analysis, the future research directions for lymphangiogenesis in breast cancer were delineated and outlined for reference. We are confident that this bibliometric research could offer a few inspirations and viable ideas for researchers in related fields.

## 6 Conclusion

Based on the published literature, this study provided a comprehensive overview of the research trend involving lymphangiogenesis in breast cancer. Since 2015, the number of relevant literature being published has declined, possibly suggesting that this research topic has reached a bottleneck. Consequently, further research, scientific investigation, and future direction are critically necessary. The US and China have the highest number of publications in this field, but the latter needs to conduct higher-quality research. Peeyush Lala and Mousumi Majumder, as highly productive authors in this field, have extensive collaborations. Journals with the most publications and citations are mostly in Q1 and Q2, demonstrating excellence in research in this area. Moreover, a keyword analysis reveals that the primary focus of research in this area is the molecular mechanism of lymphangiogenesis, with the connection between TME and lymphangiogenesis being a popular research topic in recent years and is expected to remain so in the future. To sum up, this study will afford scholars an extensive comprehension of the current state and trends in this domain, in addition to yielding ideas for future investigations.

## Author contributions

LX: Conceptualization, Formal analysis, Investigation, Methodology, Visualization, Writing – original draft, Writing – review & editing. XW: Conceptualization, Formal analysis, Investigation, Methodology, Visualization, Writing – original draft, Writing – review & editing. BW: Investigation, Software, Validation, Writing – review & editing. BM: Investigation, Validation, Writing – review & editing. XP: Conceptualization, Funding acquisition, Methodology, Writing – review & editing.
