# Supplementary material for: The global landscape and research trend of lymphangiogenesis in breast cancer: a bibliometric analysis and visualization
Source: Front Oncol. 2024 Mar 14;14:1337124. doi: 10.3389/fonc.2024.1337124 (PMC10973884; doi:10.3389/fonc.2024.1337124)
Supplement: Supplementary file 1 [file DataSheet_1.docx]

Supplementary Material

The global landscape and research trend of lymphangiogenesis in breast cancer: A bibliometric analysis and visualization

Liuyan Xu^1†^, Xuan Wang^1†^, Beibei Wang^2^, Bingxin Meng^2^, Xiaohua Pei^3*^

*** Correspondence:** Xiaohua Pei: [pxh_127@163.com](mailto:pxh_127@163.com)

# The detailed search strategy

#1 (TS=(Breast Neoplasm OR Neoplasm, Breast OR Breast Tumors OR Breast Tumor OR Tumor, Breast OR Tumors, Breast OR Neoplasms, Breast OR Breast Cancer OR Cancer, Breast OR Mammary Cancer OR Cancer, Mammary OR Cancers, Mammary OR Mammary Cancers OR Malignant Neoplasm of Breast OR Breast Malignant Neoplasm OR Breast Malignant Neoplasms OR Malignant Tumor of Breast OR Breast Malignant Tumor OR Breast Malignant Tumors OR Cancer of Breast OR Cancer of the Breast OR Mammary Carcinoma, Human OR Carcinoma, Human Mammary OR Carcinomas, Human Mammary OR Human Mammary Carcinomas OR Mammary Carcinomas, Human OR Human Mammary Carcinoma OR Mammary Neoplasms, Human OR Human Mammary Neoplasm OR Human Mammary Neoplasms OR Neoplasm, Human Mammary OR Neoplasms, Human Mammary OR Mammary Neoplasm, Human OR Breast Carcinoma OR Breast Carcinomas OR Carcinoma, Breast OR Carcinomas, Breast))

#2 TS=(Lymphangiogenesis OR Lymphangiogeneses)

#3 #1 AND #2

Date range: from inception to September 30th, 2023

Document type: Article or Review Article

# Papers included in this study

1. Kirkin V, Thiele W, Baumann P, Mazitschek R, Rohde K, Fellbrich G, et al. Maz51, an indolinone that inhibits endothelial cell and tumor cell growth in vitro, suppresses tumor growth in vivo. INT J CANCER. [Article]. 2004;112(6):986-93.

2. Van der Auwera I, Van Laere SJ, Van den Eynden GG, Benoy I, van Dam P, Colpaert CG, et al. Increased angiogenesis and lymphangiogenesis in inflammatory versus noninflammatory breast cancer by real-time reverse transcriptase-PCR gene expression quantification. CLIN CANCER RES. [Article]. 2004;10(23):7965-71.

3. Bono P, Wasenius VM, Heikkila P, Lundin J, Jackson DG, Joensuu H. High LYVE-1-positive lymphatic vessel numbers are associated with poor outcome in breast cancer. CLIN CANCER RES. [Article]. 2004;10(21):7144-9.

4. Cao RH, Bjorndahl MA, Religa P, Clasper S, Garvin S, Galter D, et al. PDGF-BB induces intratumoral lymphangiogenesis and promotes lymphatic metastasis. CANCER CELL. [Article]. 2004;6(4):333-45.

5. Bando H, Brokelmann M, Toi M, Alitalo K, Sleeman JP, Sipos B, et al. Immunodetection and quantification of vascular endothelial growth factor receptor-3 in human malignant tumor tissues. INT J CANCER. [Article]. 2004;111(2):184-91.

6. Currie MJ, Hanrahan V, Gunningham SP, Morrin HR, Frampton C, Han C, et al. Expression of vascular endothelial growth factor D is associated with hypoxia inducible factor (HIF-1 alpha) and the HIF-1 alpha target gene DEC1, but not lymph node metastasis in primary human breast carcinomas. J CLIN PATHOL. [Article]. 2004;57(8):829-34.

7. Schoppmann SF, Bayer G, Aumayr K, Taucher S, Geleff S, Rudas M, et al. Prognostic value of lymphangiogenesis and lymphovascular invasion in invasive breast cancer. ANN SURG. [Article]. 2004;240(2):306-12.

8. Jackson DG. Biology of the lymphatic marker LYVE-1 and applications in research into lymphatic trafficking and lymphangiogenesis. APMIS. [Review]. 2004;112(7-8):526-38.

9. Saharinen P, Tammela T, Karkkainen MJ, Alitalo K. Lymphatic vasculature: development, molecular regulation and role in tumor metastasis and inflammation. TRENDS IMMUNOL. [Review]. 2004;25(7):387-95.

10. Vleugel MM, Bos R, van der Groep P, Greijer AE, Shvarts A, Stel HV, et al. Lack of lymphangiogenesis during breast carcinogenesis. J CLIN PATHOL. [Article]. 2004;57(7):746-51.

11. McCarter MD, Clarke JH, Harken AH. Lymphangiogenesis is pivotal to the trials of a successful cancer metastasis. SURGERY. [Article]. 2004;135(2):121-4.

12. Saharinen P, Petrova TV. Molecular regulation of lymphangiogenesis. In: Wiedenmann B, Christofori GM, Hocker M, Reubi JC, ^editors. Annals of the New York Academy of Sciences. NEW YORK: NEW YORK ACAD SCIENCES; 2004. p. 76-87.

13. McColl BK, Loughran SJ, Davydova N, Stacker SA, Achen MG. Mechanisms of lymphangiogenesis: Targets for blocking the metastatic spread of cancer. CURR CANCER DRUG TAR. [Review]. 2005;5(8):561-71.

14. Agarwal B, Saxena R, Morimiya A, Mehrotra S, Badve S. Lymphangiogenesis does not occur in breast cancer. AM J SURG PATHOL. [Article]. 2005;29(11):1449-55.

15. Van der Auwera I, Van den Eynden GG, Colpaert CG, Van Laere SJ, van Dam P, Van Marck EA, et al. Tumor lymphangiogenesis in inflammatory breast carcinoma: A histomorphometric study. CLIN CANCER RES. [Article]. 2005;11(21):7637-42.

16. Weigelt B, Wessels L, Bosma AJ, Glas AM, Nuyten D, He YD, et al. No common denominator for breast cancer lymph node metastasis. BRIT J CANCER. [Article]. 2005;93(8):924-32.

17. Chen ZT, Varney ML, Backora MW, Cowan K, Solheim JC, Talmadge JE, et al. Down-regulation of vascular endothelial cell growth factor-C expression using small interfering RNA vectors in mammary tumors inhibits tumor lymphangiogenesis and spontaneous metastasis and enhances survival. CANCER RES. [Article]. 2005;65(19):9004-11.

18. Jennbacken K, Vallbo C, Wang WZ, Damber JE. Expression of vascular endothelial growth factor C (VEGF-C) and VEGF receptor-3 in human prostate cancer is associated with regional lymph node metastasis. PROSTATE. [Article]. 2005;65(2):110-6.

19. Leclers D, Durand K, Dutour A, Barriere G, Monteil J, Rigaud M, et al. Lymphatic vessels and cancer. M S-MED SCI. [Review]. 2005;21(10):839-47.

20. Al-Rawi M, Watkins G, Mansel RE, Jiang WG. The effects of interleukin-7 on the lymphangiogenic properties of human endothelial cells. INT J ONCOL. [Article]. 2005;27(3):721-30.

21. Cao YH. Opinion - Emerging mechanisms of tumour lymphangiogenesis and lymphatic metastasis. NAT REV CANCER. [Review]. 2005;5(9):735-43.

22. Guo LX, Zou K, Ju JH, Xie H. Hyaluronan promotes tumor lymphangiogenesis and intralymphantic tumor growth in xenografts. ACTA BIOCH BIOPH SIN. [Article]. 2005;37(9):601-6.

23. Okada K, Osaki M, Araki K, Ishiguro K, Ito H, Ohgi S. Expression of hypoxia-inducible factor (HIF-1 alpha), VEGF-C and VEGF-D in non-invasive and invasive breast ductal carcinomas. ANTICANCER RES. [Article]. 2005;25(4):3003-9.

24. Yavuz S, Paydas S, Disel U, Zorludemir S, Erdogan S. VEGF-C expression in breast cancer: Clinical importance. ADV THER. [Article]. 2005;22(4):368-80.

25. Wulfing P, Kersting C, Buerger H, Mattsson B, Mesters R, Gustmann C, et al. Expression patterns of angiogenic and lymphangiogenic factors in ductal breast carcinoma in situ. BRIT J CANCER. [Article]. 2005;92(9):1720-8.

26. Nakamura Y, Yasuoka H, Tsujimoto M, Imabun S, Nakahara M, Nakao K, et al. Lymph vessel density correlates with nodal status, VEGF-C expression, and prognosis in breast cancer. BREAST CANCER RES TR. [Article]. 2005;91(2):125-32.

27. Akahane M, Akahane T, Shah A, Okajima E, Thorgeirsson UP. A potential role for vascular endothelial growth factor-D as an autocrine growth factor for human breast carcinoma cells. ANTICANCER RES. [Article]. 2005;25(2A):701-7.

28. Al-Rawi M, Watkins G, Mansel RE, Jiang WG. Interleukin 7 upregulates vascular endothelial growth factor D in breast cancer cells and induces lymphangiogenesis in vivo. BRIT J SURG. [Article]. 2005;92(3):305-10.

29. Koukourakis MI, Giatromanolaki A, Sivridis E, Simopoulos C, Gatter KC, Harris AL, et al. LYVE-1 immunohistochemical assessment of lymphangiogenesis in endometrial and lung cancer. J CLIN PATHOL. [Article]. 2005;58(2):202-6.

30. Pytowski B, Goldman J, Persaud K, Wu Y, Witte L, Hicklin DJ, et al. Complete and specific inhibition of adult lymphatic regeneration by a novel VEGFR-3 neutralizing antibody. JNCI-J NATL CANCER I. [Article]. 2005;97(1):14-21.

31. Choi W, Lewis MM, Lawson D, Yin-Goen QQ, Birdsong GG, Cotsonis GA, et al. Angiogenic and lymphangiogenic microvessel density in breast carcinoma: correlation with clinicopathologic parameters and VEGF-family gene expression. MODERN PATHOL. [Article]. 2005;18(1):143-52.

32. Filho AL, Martins A, Costa S, Schmitt FC. VEGFR-3 expression in breast cancer tissue is not restricted to lymphatic vessels. PATHOL RES PRACT. [Article]. 2005;201(2):93-9.

33. Vantyghem SA, Allan AL, Postenka CO, Al-Katib W, Keeney M, Tuck AB, et al. A new model for lymphatic metastasis: Development of a variant of the MDA-MB-468 human breast cancer cell line that aggressively metastasizes to lymph nodes. CLIN EXP METASTAS. [Article]. 2005;22(4):351-61.

34. Wilting J, Hawighorst T, Hecht M, Christ B, Papoutsi M. Development of lymphatic vessels: Tumour lymphangiogenesis and lymphatic invasion. CURR MED CHEM. [Review]. 2005;12(26):3043-53.

35. Van der Auwera I, Cao Y, Tille JC, Pepper MS, Jackson DG, Fox SB, et al. First international consensus on the methodology of lymphangiogenesis quantification in solid human tumours. BRIT J CANCER. [Review]. 2006;95(12):1611-25.

36. Ji R. Lymphatic endothelial cells, tumor lymphangiogenesis and metastasis: New insights into intratumoral and peritumoral lymphatics. CANCER METAST REV. [Review]. 2006;25(4):677-94.

37. Van den Eynden GG, Van der Auwera I, Van Laere SJ, Huygelen V, Colpaert CG, van Dam P, et al. Induction of lymphangiogenesis in and around axillary lymph node metastases of patients with breast cancer. BRIT J CANCER. [Article]. 2006;95(10):1362-6.

38. Qian C, Berghuis B, Tsarfaty G, Bruch M, Kort EJ, Ditlev J, et al. Preparing the "soil": The primary tumor induces vasculature reorganization in the sentinel lymph node before the arrival of metastatic cancer cells. CANCER RES. [Article]. 2006;66(21):10365-76.

39. Harrell JC, Dye WW, Allred DC, Jedlicka P, Spoelstra NS, Sartorius CA, et al. Estrogen receptor positive breast cancer metastasis: Altered hormonal sensitivity and tumor aggressiveness in lymphatic vessels and lymph nodes. CANCER RES. [Article]. 2006;66(18):9308-15.

40. Hulit J, Lee RJ, Li Z, Wang C, Katiyar S, Yang J, et al. p27(Kip1) repression of ErbB2-induced mammary tumor growth in transgenic mice involves Skp2 and Wnt/beta-catenin signaling. CANCER RES. [Article]. 2006;66(17):8529-41.

41. Schoppmann SF, Fenzl A, Schindl M, Bachleitner-Hofmann T, Nagy K, Gnant M, et al. Hypoxia inducible factor-1 alpha correlates with VEGF-C expression and lymphangiogenesis in breast cancer. BREAST CANCER RES TR. [Article]. 2006;99(2):135-41.

42. Shayan R, Achen MG, Stacker SA. Lymphatic vessels in cancer metastasis: bridging the gaps. CARCINOGENESIS. [Review]. 2006;27(9):1729-38.

43. Allan AL, George R, Vantyghem SA, Lee MW, Hodgson NC, Engel CJ, et al. Role of the integrin-binding protein osteopontin in lymphatic metastasis of breast cancer. AM J PATHOL. [Article]. 2006;169(1):233-46.

44. Schoppmann SF, Fenzl A, Nagy K, Unger S, Bayer G, Geleff S, et al. VEGF-C expressing tumor-associated macrophages in lymphnode positive breast cancer: impact on lymphangiogenesis and survival. SURGERY. [Article]. 2006;139(6):839-46.

45. Leclers D, Durand K, Cook-Moreau J, Rabinovitch-Chable H, Sturtz FG, Rigaud M. VEGFR-3, VEGF-C and VEGF-D mRNA quantification by RT-PCR in different human cell types. ANTICANCER RES. [Article]. 2006;26(3A):1885-91.

46. Li YS, Kaneko M, Amatya VJ, Takeshima Y, Arihiro K, Inai K. Expression of vascular endothelial growth factor-C and its receptor in invasive micropapillary carcinoma of the breast. PATHOL INT. [Article]. 2006;56(5):256-61.

47. Timoshenko AV, Chakraborty C, Wagner GF, Lala PK. COX-2-mediated stimulation of the lymphangiogenic factor VEGF-C in human breast cancer. BRIT J CANCER. [Article]. 2006;94(8):1154-63.

48. Bando H, Weich HA, Horiguchi S, Funata N, Ogawa T, Toi M. The association between vascular endothelial growth factor-C, its corresponding receptor, VEGFR-3, and prognosis in primary breast cancer: A study with 193 cases. ONCOL REP. [Article]. 2006;15(3):653-9.

49. Su JL, Yang PC, Shih JY, Yang CY, Wei LH, Hsieh CY, et al. The VEGF-C/Flt-4 axis promotes invasion and metastasis of cancer cells. CANCER CELL. [Article]. 2006;9(3):209-23.

50. Nakamura Y, Yasuoka H, Tsujimoto M, Yoshidome K, Nakahara M, Nakao K, et al. Nitric oxide in breast cancer: Induction of vascular endothelial growth factor-C and correlation with metastasis and poor prognosis. CLIN CANCER RES. [Article]. 2006;12(4):1201-7.

51. Stacker SA, Hughes RA, Williams RA, Achen MG. Current strategies for modulating lymphangiogenesis signalling pathways in human disease. CURR MED CHEM. [Review]. 2006;13(7):783-92.

52. Whitehurst B, Flister MJ, Bagaitkar J, Volk L, Bivens CM, Pickett B, et al. Anti-VEGF-A therapy reduces lymphatic vessel density and expression of VEGFR-3 in an orthotopic breast tumor model. INT J CANCER. [Article]. 2007;121(10):2181-91.

53. Eccles S, Paon L, Sleeman J. Lymphatic metastasis in breast cancer: importance and new insights into cellular and molecular mechanisms. CLIN EXP METASTAS. [Review]. 2007;24(8):619-36.

54. van Iterson V, Leidenius M, von Smitten K, Bono P, Heikkila P. VEGF-D in association with VEGFR-3 promotes nodal metastasis in human invasive lobular breast cancer. AM J CLIN PATHOL. [Article]. 2007;128(5):759-66.

55. Yu M, Tang Z, Alousi S, Berk RS, Miller F, Kosir MA. Expression patterns of lymphangio genic and angiogenic factors in a model of breast ductal carcinoma in situ. AM J SURG. [Article; Proceedings Paper]. 2007;194(5):594-9.

56. Timoshenko AV, Rastogi S, Lala PK. Migration-promoting role of VEGF-C and VEGF-C binding receptors in human breast cancer cells. BRIT J CANCER. [Article]. 2007;97(8):1090-8.

57. Van den Eynden GG, Colpaert CG, Couvelard A, Pezzella F, Dirix LY, Vermeulen PB, et al. A fibrotic focus is a prognostic factor and a surrogate marker for hypoxia and (lymph)angiogenesis in breast cancer: review of the literature and proposal on the criteria of evaluation. HISTOPATHOLOGY. [Review]. 2007;51(4):440-51.

58. Sundar SS, Ganesan TS. Role of lymphangiogenesis in cancer. J CLIN ONCOL. [Review]. 2007;25(27):4298-307.

59. Van den Eynden GG, Vandenberghe MK, van Dam PH, Colpaert CG, van Dam P, Dirix LY, et al. Increased sentinel lymph node lymphangiogenesis is associated with nonsentinel axillary lymph node involvement in breast cancer patients with a positive sentinel node. CLIN CANCER RES. [Article]. 2007;13(181):5391-7.

60. Van den Eynden GG, Van der Auwera I, Van Laere SJ, Trinh XB, Colpaert CG, van Dam P, et al. Comparison of molecular determinants of angiogenesis and lymphangiogenesis in lymph node metastases and in primary tumours of patients with breast cancer. J PATHOL. [Article]. 2007;213(1):56-64.

61. van der Schaft DWJ, Pauwels P, Hulsmans S, Zimmermann M, de Poll-Franse LVV, Griffioen AW. Absence of lymphangiogenesis in ductal breast cancer at the primary tumor site. CANCER LETT. [Article]. 2007;254(1):128-36.

62. Liersch R, Detmar M. Lymphangiogenesis in development and disease. THROMB HAEMOSTASIS. [Article]. 2007;98(2):304-10.

63. Mylona E, Nomikos A, Alexandrou P, Giannopoulou I, Keramopoulos A, Nakopoulou L. Lymphatic and blood vessel morphometry in invasive breast carcinomas: Relation with proliferation and VEGF-C and -D proteins expression. HISTOL HISTOPATHOL. [Article]. 2007;22(8):825-35.

64. Eichten A, Hyun WC, Coussens LM. Distinctive features of anglogenesis and lymphangiogenesis determine their functionality during de novo tumor development. CANCER RES. [Article]. 2007;67(11):5211-20.

65. Al-Mowallad A, Kirwan C, Byrne G, McDowell G, Li C, Stewart A, et al. Vascular endothelial growth factor-C in patients with breast cancer. IN VIVO. [Article]. 2007;21(3):549-51.

66. Mohammed RAA, Green A, El-Shikh S, Paish EC, Ellis IO, Martin SG. Prognostic significance of vascular endothelial cell growth factors -A, -C and -D in breast cancer and their relationship with angio- and lymphangiogenesis. BRIT J CANCER. [Article]. 2007;96(7):1092-100.

67. Barnes NLP, Warnberg F, Farnie G, White D, Jiang W, Anderson E, et al. Cyclooxygenase-2 inhibition: effects on tumour growth, cell cycling and lymphangiogenesis in a xenograft model of breast cancer. BRIT J CANCER. [Article]. 2007;96(4):575-82.

68. Su J, Yen C, Chen P, Chuang S, Hong C, Kuo I, et al. The role of the VEGF-C/VEGFR-3 axis in cancer progression. BRIT J CANCER. [Review]. 2007;96(4):541-5.

69. Arnaout-Alkarain A, Kahn HJ, Narod SA, Sun PA, Marks AN. Significance of lymph vessel invasion identified by the endothelial lymphatic marker D2-40 in node negative breast cancer. MODERN PATHOL. [Article]. 2007;20(2):183-91.

70. Sarli G, Sassi F, Brunetti B, Rizzo A, Diracca L, Benazzi C. Lymphatic vessels assessment in feline mammary tumours. BMC CANCER. [Article]. 2007;7(7).

71. Garmy-Susini B, Makale M, Fuster M, Varner JA. Methods to study lymphatic vessel integrins. In: Cheresh DA, editor Methods in Enzymology. SAN DIEGO: ELSEVIER ACADEMIC PRESS INC; 2007. p. 415-38.

72. Goette M, Kersting C, Radke I, Kiesel L, Wuelfing P. An expression signature of syndecan-1 (CD138), E-cadherin and c-met is associated with factors of angiogenesis and lymphangiogenesis in ductal breast carcinoma in situ. BREAST CANCER RES. [Article]. 2007;9(R81).

73. Cohen-Kaplan V, Naroditsky I, Zetser A, Illan N, Vlodavsky I, Doweck I. Heparanase induces VEGF C and facilitates tumor lymphangiogenesis. INT J CANCER. [Article]. 2008;123(11):2566-73.

74. Jakab C, Halasz J, Kiss A, Schaff Z, Szasz AM, Rusvai M, et al. EVALUATION OF MICROVESSEL DENSITY (MVD) IN CANINE MAMMARY TUMOURS BY QUANTITATIVE IMMUNOHISTOCHEMISTRY OF THE CLAUDIN-5 MOLECULE. ACTA VET HUNG. [Article]. 2008;56(4):495-510.

75. Shibata M, Morimoto J, Shibata E, Otsuki Y. Combination therapy with short interfering RNA vectors against VEGF-C and VEGF-A suppresses lymph node and lung metastasis in a mouse immunocompetent mammary cancer model. CANCER GENE THER. [Article]. 2008;15(12):776-86.

76. Gu Y, Qi X, Guo S. Lymphangiogenesis induced by VEGF-C and VEGF-D promotes metastasis and a poor outcome in breast carcinoma: a retrospective study of 61 cases. CLIN EXP METASTAS. [Article]. 2008;25(7):717-25.

77. Bednarek W, Wertel I, Kotarski J. Lymphangiogenesis in cancerous tumours. GINEKOL POL. [Article]. 2008;79(9):625-9.

78. Lin CI, Chen CN, Huang MT, Lee SJ, Lin CH, Chang CC, et al. Lysophosphatidic acid up-regulates vascular endothelial growth factor-C and lymphatic marker expressions in human endothelial cells. CELL MOL LIFE SCI. [Article]. 2008;65(17):2740-51.

79. Matsui J, Funahashi Y, Uenaka T, Watanabe T, Tsuruoka A, Asada M. Multi-kinase inhibitor E7080 suppresses lymph node and lung metastases of human mammary breast tumor MDA-MB-231 via inhibition of vascular endothelial growth factor-receptor (VEGF-R) 2 and VEGF-R3 kinase. CLIN CANCER RES. [Article]. 2008;14(17):5459-65.

80. Banziger-Tobler NE, Halin C, Kajiya K, Detmar M. Growth hormone promotes lymphangiogenesis. AM J PATHOL. [Article]. 2008;173(2):586-97.

81. Ruddell A, Kelly-Spratt KS, Furuya M, Parghi SS, Kemp CJ. p19/Arf and p53 suppress sentinel lymph node lymphangiogenesis and carcinoma metastasis. ONCOGENE. [Article]. 2008;27(22):3145-55.

82. El-Gohary YM, Metwally G, Saad RS, Robinson MJ, Mesko T, Poppiti RJ. Prognostic significance of intratumoral and peritumoral lymphatic density and blood vessel density in invasive breast carcinomas. AM J CLIN PATHOL. [Article; Proceedings Paper]. 2008;129(4):578-86.

83. Mikhaylova M, Mori N, Wildes FB, Walczak P, Gimi B, Bhujwalla ZM. Hypoxia increases breast cancer cell-induced lymphatic endothelial cell migration. NEOPLASIA. [Article]. 2008;10(4):380-5.

84. Cunnick GH, Jiang WG, Douglas-Jones T, Watkins G, Gomez KF, Morgan MJ, et al. Lymphangiogenesis and lymph node metastasis in breast cancer. MOL CANCER. [Article]. 2008;7(23).

85. Marinho VFZ, Metze K, Sanches FSF, Rocha GFS, Gobbi H. Lymph vascular invasion in invasive mammary carcinomas identified by the endothelial lymphatic marker D2-40 is associated with other indicators of poor prognosis. BMC CANCER. [Article]. 2008;8(64).

86. Zhang X, Huang D, Guo G, Chen G, Zhang H, Wan L, et al. Coexpression of VEGF-C and COX-2 and its association with lymphangiogenesis in human breast cancer. BMC CANCER. [Article]. 2008;8(4).

87. Karpanen T, Alitalo K. Molecular biology and pathology of lymphangiogenesis. ANNU REV PATHOL-MECH. [Review; Book Chapter]. 2008;3:367-97.

88. Koyama H, Kobayashi N, Harada M, Takeoka M, Kawai Y, Sano K, et al. Significance of tumor-associated stroma in promotion of intratumoral lymphangiogenesis - Pivotal role of a hyaluronan-rich tumor microenvironment. AM J PATHOL. [Article]. 2008;172(1):179-93.

89. Laakkonen P, Zhang L, Ruoslahti E. Peptide targeting of tumor lymph vessels. LYMPHATIC CONTINUUM REVISITED. [Article]. 2008;1131:37-43.

90. Sun P, Gao J, Liu Y, Wei L, Wu L, Liu Z. RNA interference (RNAi)-mediated vascular endothelial growth factor-C (VEGF-C) reduction interferes with lymphangiogenesis and enhances Epirubicin sensitivity of breast cancer cells. MOL CELL BIOCHEM. [Article]. 2008;308(1-2):161-8.

91. Sleeman JP, Thiele W. Tumor metastasis and the lymphatic vasculature. INT J CANCER. [Review]. 2009;125(12):2747-56.

92. Liu H, Ma R, Yang Q, Du G, Zhang C. Lymphangiogenic Characteristics of Triple Negativity in Node-Negative Breast Cancer. INT J SURG PATHOL. [Article]. 2009;17(6):426-31.

93. Yang Z, Adams AL, Hameed O. Attenuated Podoplanin Staining in Breast Myoepithelial Cells A Potential Caveat in the Diagnosis of Lymphatic Invasion. APPL IMMUNOHISTO M M. [Article]. 2009;17(5):425-30.

94. Cohen B, Addadi Y, Sapoznik S, Meir G, Kalchenko V, Harmelin A, et al. Transcriptional Regulation of Vascular Endothelial Growth Factor C by Oxidative and Thermal Stress Is Mediated by Lens Epithelium-Derived Growth Factor/p75. NEOPLASIA. [Article]. 2009;11(9):271-921.

95. Gu X, Cao Y. Inhibition effect of shRNA on VEGF-C in breast cancer cells. CHINESE J CANCER RES. [Article]. 2009;21(3):202-6.

96. Boneberg E, Legler DF, Hoefer MM, Oehlschlegel C, Steininger H, Fuezesi L, et al. Angiogenesis and lymphangiogenesis are downregulated in primary breast cancer. BRIT J CANCER. [Article]. 2009;101(4):605-14.

97. Mumprecht V, Detmar M. Lymphangiogenesis and cancer metastasis. J CELL MOL MED. [Review]. 2009;13(8A):1405-16.

98. Hirakawa S. From tumor lymphangiogenesis to lymphvascular niche. CANCER SCI. [Review]. 2009;100(6):983-9.

99. Kubota Y, Takubo K, Shimizu T, Ohno H, Kishi K, Shibuya M, et al. M-CSF inhibition selectively targets pathological angiogenesis and lymphangiogenesis. J EXP MED. [Article]. 2009;206(5):1089-102.

100. Dunworth WP, Caron KM. G Protein-Coupled Receptors as Potential Drug Targets for Lymphangiogenesis and Lymphatic Vascular Diseases. ARTERIOSCL THROM VAS. [Review]. 2009;29(5):650-6.

101. Guo B, Zhang Y, Luo G, Li L, Zhang J. Lentivirus-Mediated Small Interfering RNA Targeting VEGF-C Inhibited Tumor Lymphangiogenesis and Growth in Breast Carcinoma. ANAT REC. [Article]. 2009;292(5):633-9.

102. Huyn ST, Burton JB, Sato M, Carey M, Gambhir SS, Wu L. A Potent, Imaging Adenoviral Vector Driven by the Cancer-selective Mucin-1 Promoter That Targets Breast Cancer Metastasis. CLIN CANCER RES. [Article]. 2009;15(9):3126-34.

103. Britto AV, Schenka AA, Moraes-Schenka NG, Alvarenga M, Shinzato JY, Vassallo J, et al. Immunostaining with D2-40 improves evaluation of lymphovascular invasion, but may not predict sentinel lymph node status in early breast cancer. BMC CANCER. [Article]. 2009;9(109).

104. Marneros AG, Blanco F, Husain S, Silvers DN, Grossman ME. Classification of cutaneous intravascular breast cancer metastases based on immunolabeling for blood and lymph vessels. J AM ACAD DERMATOL. [Article]. 2009;60(4):633-8.

105. Nagasaki S, Suzuki T, Miki Y, Akahira J, Shibata H, Ishida T, et al. Chicken ovalbumin upstream promoter transcription factor II in human breast carcinoma: Possible regulator of lymphangiogenesis via vascular endothelial growth factor-C expression. CANCER SCI. [Article]. 2009;100(4):639-45.

106. Spinella F, Garrafa E, Di Castro V, Rosano L, Nicotra MR, Caruso A, et al. Endothelin-1 Stimulates Lymphatic Endothelial Cells and Lymphatic Vessels to Grow and Invade. CANCER RES. [Article]. 2009;69(6):2669-76.

107. Brunet-Dunand SE, Vouyovitch C, Araneda S, Pandey V, Vidal LJP, Print C, et al. Autocrine Human Growth Hormone Promotes Tumor Angiogenesis in Mammary Carcinoma. ENDOCRINOLOGY. [Article]. 2009;150(3):1341-52.

108. Ji RC. Lymph node lymphangiogenesis: a new concept for modulating tumor metastasis and inflammatory process. HISTOL HISTOPATHOL. [Review]. 2009;24(3):377-84.

109. de Mascarel I, MacGrogan G, Debled M, Sierankowski G, Brouste V, Mathoulin-Pelissier S, et al. D2-40 in breast cancer: should we detect more vascular emboli? MODERN PATHOL. [Article]. 2009;22(2):216-22.

110. Mohammed RAA, Ellis IO, Elsheikh S, Paish EC, Martin SG. Lymphatic and angiogenic characteristics in breast cancer: morphometric analysis and prognostic implications. BREAST CANCER RES TR. [Article]. 2009;113(2):261-73.

111. Utrera-Barillas MD, Castro-Manrreza ME, Gutierrez-Rodriguez M, Benitez-Bribiesca L. Cancer lymphangiogenesis and its role in metastasic dissemination. GAC MED MEX. [Review]. 2009;145(1):51-60.

112. Shibata M, Ambati J, Shibata E, Albuquerque RJC, Morimoto J, Ito Y, et al. The endogenous soluble VEGF receptor-2 isoform suppresses lymph node metastasis in a mouse immunocompetent mammary cancer model. BMC MED. [Article]. 2010;8(69).

113. Acs G, Esposito NN, Rakosy Z, Laronga C, Zhang PJ. Invasive Ductal Carcinomas of the Breast Showing Partial Reversed Cell Polarity are Associated With Lymphatic Tumor Spread and may Represent Part of a Spectrum of Invasive Micropapillary Carcinoma. AM J SURG PATHOL. [Article]. 2010;34(11):1637-46.

114. Mumprecht V, Honer M, Vigl B, Proulx ST, Trachsel E, Kaspar M, et al. In vivo Imaging of Inflammation- and Tumor-Induced Lymph Node Lymphangiogenesis by Immuno-Positron Emission Tomography. CANCER RES. [Article]. 2010;70(21):8842-51.

115. Zhuo W, Luo C, Wang X, Song X, Fu Y, Luo Y. Endostatin inhibits tumour lymphangiogenesis and lymphatic metastasis via cell surface nucleolin on lymphangiogenic endothelial cells. J PATHOL. [Article]. 2010;222(3):249-60.

116. Kitano H, Kageyama S, Hewitt SM, Hayashi R, Doki Y, Ozaki Y, et al. Podoplanin Expression in Cancerous Stroma Induces Lymphangiogenesis and Predicts Lymphatic Spread and Patient Survival. ARCH PATHOL LAB MED. [Article]. 2010;134(10):1520-7.

117. Wiig H, Keskin D, Kalluri R. Interaction between the extracellular matrix and lymphatics: Consequences for lymphangiogenesis and lymphatic function. MATRIX BIOL. [Review]. 2010;29(8):645-56.

118. Bhattacharjee RN, Timoshenko AV, Cai J, Lala PK. Relationship between cyclooxygenase-2 and human epidermal growth factor receptor 2 in vascular endothelial growth factor C up-regulation and lymphangiogenesis in human breast cancer. CANCER SCI. [Article]. 2010;101(9):2026-32.

119. Lee SK, Cho EY, Kim WW, Kim SH, Hur SM, Kim S, et al. The Prediction of Lymph Node Metastasis in Ductal Carcinoma In Situ With Microinvasion by Assessing Lymphangiogenesis. J SURG ONCOL. [Article]. 2010;102(3):225-9.

120. Anelli V, Gault CR, Snider AJ, Obeid LM. Role of sphingosine kinase-1 in paracrine/transcellular angiogenesis and lymphangiogenesis in vitro. FASEB J. [Article]. 2010;24(8):2727-38.

121. Cueni LN, Hegyi I, Shin JW, Albinger-Hegyi A, Gruber S, Kunstfeld R, et al. Tumor Lymphangiogenesis and Metastasis to Lymph Nodes Induced by Cancer Cell Expression of Podoplanin. AM J PATHOL. [Article]. 2010;177(2):1004-16.

122. Ischenko I, Seeliger H, Camaj P, Kleespies A, Guba M, Eichhorn ME, et al. Src Tyrosine Kinase Inhibition Suppresses Lymphangiogenesis In Vitro and In Vivo. CURR CANCER DRUG TAR. [Review]. 2010;10(5):546-53.

123. Larrieu-Lahargue F, Welm AL, Thomas KR, Li DY. Netrin-4 induces lymphangiogenesis in vivo. BLOOD. [Article]. 2010;115(26):5418-26.

124. Vermeulen PB, van Golen KL, Dirix LY. Angiogenesis, Lymphangiogenesis, Growth Pattern, and Tumor Emboli in Inflammatory Breast Cancer. CANCER-AM CANCER SOC. [Article; Proceedings Paper]. 2010;116S(11):2748-54.

125. Wu M, Han L, Shi Y, Xu G, Wei J, You L, et al. Development and characterization of a novel method for the analysis of gene expression patterns in lymphatic endothelial cells derived from primary breast tissues. J CANCER RES CLIN. [Article]. 2010;136(6):863-72.

126. Schoppmann SF, Tamandl D, Roberts L, Jomrich G, Schoppmann A, Zwrtek R, et al. HER2/neu expression correlates with vascular endothelial growth factor-C and lymphangiogenesis in lymph node-positive breast cancer. ANN ONCOL. [Article]. 2010;21(5):955-60.

127. Garmy-Susini B, Avraamides CJ, Schmid MC, Foubert P, Ellies LG, Barnes L, et al. Integrin alpha 4 beta 1 Signaling Is Required for Lymphangiogenesis and Tumor Metastasis. CANCER RES. [Article]. 2010;70(8):3042-51.

128. Bruyere F, Melen-Lamalle L, Blacher S, Detry B, Masset A, Lecomte J, et al. Does Plasminogen Activator Inhibitor-1 Drive Lymphangiogenesis? PLOS ONE. [Article]. 2010;5(e96533).

129. Raica M, Ribatti D. Targeting Tumor Lymphangiogenesis: An Update. CURR MED CHEM. [Review]. 2010;17(8):698-708.

130. Tsutsui S, Matsuyama A, Yamamoto M, Takeuchi H, Oshiro Y, Ishida T, et al. The Akt expression correlates with the VEGF-A and -C expression as well as the microvessel and lymphatic vessel density in breast cancer. ONCOL REP. [Article]. 2010;23(3):621-30.

131. Tammela T, Alitalo K. Lymphangiogenesis: Molecular Mechanisms and Future Promise. CELL. [Review]. 2010;140(4):460-76.

132. Kubo H, Hosono K, Suzuki T, Ogawa Y, Kato H, Kamata H, et al. Host prostaglandin EP3 receptor signaling relevant to tumor-associated lymphangiogenesis. BIOMED PHARMACOTHER. [Article]. 2010;64(2):101-6.

133. Yan A, Avraham T, Zampell JC, Haviv YS, Weitman E, Mehrara BJ. Adipose-derived stem cells promote lymphangiogenesis in response to VEGF-C stimulation or TGF-beta 1 inhibition. FUTURE ONCOL. [Article]. 2011;7(12):1457-73.

134. Kashiwagi S, Hosono K, Suzuki T, Takeda A, Uchinuma E, Majima M. Role of COX-2 in lymphangiogenesis and restoration of lymphatic flow in secondary lymphedema. LAB INVEST. [Article]. 2011;91(9):1314-25.

135. Harris NC, Paavonen K, Davydova N, Roufail S, Sato T, Zhang Y, et al. Proteolytic processing of vascular endothelial growth factor-D is essential for its capacity to promote the growth and spread of cancer. FASEB J. [Article]. 2011;25(8):2615-25.

136. Lee AS, Kim DH, Lee JE, Jung YJ, Kang KP, Lee S, et al. Erythropoietin Induces Lymph Node Lymphangiogenesis and Lymph Node Tumor Metastasis. CANCER RES. [Article]. 2011;71(13):4506-17.

137. Kerjaschki D, Bago-Horvath Z, Rudas M, Sexl V, Schneckenleithner C, Wolbank S, et al. Lipoxygenase mediates invasion of intrametastatic lymphatic vessels and propagates lymph node metastasis of human mammary carcinoma xenografts in mouse. J CLIN INVEST. [Article]. 2011;121(5):2000-12.

138. Raica M, Cimpean AM, Ceausu R, Ribatti D. Lymphatic Microvessel Density, VEGF-C, and VEGFR-3 Expression in Different Molecular Types of Breast Cancer. ANTICANCER RES. [Article]. 2011;31(5):1757-64.

139. Witte MH, Dellinger MT, McDonald DM, Nathanson SD, Boccardo FM, Campisi CCC, et al. Lymphangiogenesis and Hemangiogenesis: Potential Targets for Therapy. J SURG ONCOL. [Article]. 2011;103(6SI):489-500.

140. Timoshenko AV. Chitin hydrolysate stimulates VEGF-C synthesis by MDA-MB-231 breast cancer cells. CELL BIOL INT. [Article]. 2011;35(3):281-6.

141. Tammela T, Saaristo A, Holopainen T, Yla-Herttuala S, Andersson LC, Virolainen S, et al. Photodynamic Ablation of Lymphatic Vessels and Intralymphatic Cancer Cells Prevents Metastasis. SCI TRANSL MED. [Article]. 2011;3(69ra1169).

142. Hirakawa S. Regulation of pathological lymphangiogenesis requires factors distinct from those governing physiological lymphangiogenesis. J DERMATOL SCI. [Review]. 2011;61(2):85-93.

143. Mohammed RAA, Martin SG, Mahmmod AM, Macmillan RD, Green AR, Paish EC, et al. Objective assessment of lymphatic and blood vascular invasion in lymph node-negative breast carcinoma: findings from a large case series with long-term follow-up. J PATHOL. [Article]. 2011;223(3):358-65.

144. Nakao S, Zandi S, Hata Y, Kawahara S, Arita R, Schering A, et al. Blood vessel endothelial VEGFR-2 delays lymphangiogenesis: an endogenous trapping mechanism links lymph- and angiogenesis. BLOOD. [Article]. 2011;117(3):1081-90.

145. Kodera Y, Katanasaka Y, Kitamura Y, Tsuda H, Nishio K, Tamura T, et al. Sunitinib inhibits lymphatic endothelial cell functions and lymph node metastasis in a breast cancer model through inhibition of vascular endothelial growth factor receptor 3. BREAST CANCER RES. [Article]. 2011;13(R663).

146. Ribatti D, Crivellato E. MAST CELLS, ANGIOGENESIS AND CANCER. MAST CELL BIOLOGY: CONTEMPORARY AND EMERGING TOPICS. [Article; Book Chapter]. 2011;716:270-88.

147. Yoon SY, Lee HR, Park Y, Kim JH, Kim SY, Yoon SR, et al. Thymosin beta 4 expression correlates with lymph node metastasis through hypoxia inducible factor-alpha induction in breast cancer. ONCOL REP. [Article]. 2011;25(1):23-31.

148. Betterman KL, Paquet-Fifield S, Asselin-Labat M, Visvader JE, Butler LM, Stacker SA, et al. Remodeling of the Lymphatic Vasculature during Mouse Mammary Gland Morphogenesis Is Mediated via Epithelial-Derived Lymphangiogenic Stimuli. AM J PATHOL. [Article]. 2012;181(6):2225-38.

149. Liersch R, Hirakawa S, Berdel WE, Mesters RM, Detmar M. Induced lymphatic sinus hyperplasia in sentinel lymph nodes by VEGF-C as the earliest premetastatic indicator. INT J ONCOL. [Article]. 2012;41(6):2073-8.

150. Shibata M, Ambati J, Shibata E, Yoshidome K, Harada-Shiba M. Mammary cancer gene therapy targeting lymphangiogenesis: VEGF-C siRNA and soluble VEGF receptor-2, a splicing variant. MED MOL MORPHOL. [Review]. 2012;45(4):179-84.

151. Wang J, Guo Y, Wang B, Bi J, Li K, Liang X, et al. Lymphatic microvessel density and vascular endothelial growth factor-C and -D as prognostic factors in breast cancer: a systematic review and meta-analysis of the literature. MOL BIOL REP. [Article]. 2012;39(12):11153-65.

152. Zhao Y, Ni X, Wang M, Zha X, Zhao Y, Wang S. Tumor-derived VEGF-C, but not VEGF-D, promotes sentinel lymph node lymphangiogenesis prior to metastasis in breast cancer patients. MED ONCOL. [Article]. 2012;29(4):2594-600.

153. Zampell JC, Yan A, Elhadad S, Avraham T, Weitman E, Mehrara BJ. CD4+Cells Regulate Fibrosis and Lymphangiogenesis in Response to Lymphatic Fluid Stasis. PLOS ONE. [Article]. 2012;7(e4994011).

154. Chen Y, Zhu C, Peng Z, Dai Y, Gu Y. Lentivirus-mediated short-hairpin RNA targeting IGF-1R inhibits growth and lymphangiogenesis in breast cancer. ONCOL REP. [Article]. 2012;28(5):1778-84.

155. Ding M, Fu X, Tan H, Wang R, Chen Z, Ding S. The effect of vascular endothelial growth factor C expression in tumor-associated macrophages on lymphangiogenesis and lymphatic metastasis in breast cancer. MOL MED REP. [Article]. 2012;6(5):1023-9.

156. Niemiec J, Adamczyk A, Ambicka A, Mucha-Malecka A, Wysocki W, Mitus J, et al. LYMPHANGIOGENESIS ASSESSMENT AND ITS RELATION TO TUMOUR GRADE, BREAST CANCER SUBTYPE AND EXPRESSION OF BASAL MARKERS. POL J PATHOL. [Article]. 2012;63(3):165-71.

157. Yiannakopoulou E. Modulation of Lymphangiogenesis: A New Target for Aspirin and Other Nonsteroidal Anti-inflammatory Agents? A Systematic Review. J CLIN PHARMACOL. [Review]. 2012;52(11):1749-54.

158. Schito L, Rey S, Tafani M, Zhang H, Wong CC, Russo A, et al. Hypoxia-inducible factor 1-dependent expression of platelet-derived growth factor B promotes lymphatic metastasis of hypoxic breast cancer cells. P NATL ACAD SCI USA. [Article]. 2012;109(40):E2707-16.

159. Alitalo A, Detmar M. Interaction of tumor cells and lymphatic vessels in cancer progression. ONCOGENE. [Review]. 2012;31(42):4499-508.

160. Zhuo W, Jia L, Song N, Lu X, Ding Y, Wang X, et al. The CXCL12-CXCR4 Chemokine Pathway: A Novel Axis Regulates Lymphangiogenesis. CLIN CANCER RES. [Article]. 2012;18(19):5387-98.

161. Wang Z, Yu Y, Ma J, Zhang H, Zhang H, Wang X, et al. LyP-1 Modification To Enhance Delivery of Artemisinin or Fluorescent Probe Loaded Polymeric Micelles to Highly Metastatic Tumor and Its Lymphatics. MOL PHARMACEUT. [Article]. 2012;9(9):2646-57.

162. Zhao Y, Ni X, Li Y, Dai M, Yuan Z, Zhu Y, et al. Peritumoral lymphangiogenesis induced by vascular endothelial growth factor C and D promotes lymph node metastasis in breast cancer patients. WORLD J SURG ONCOL. [Article]. 2012;10(165).

163. Alam A, Blanc I, Gueguen-Dorbes G, Duclos O, Bonnin J, Barron P, et al. SAR131675, a Potent and Selective VEGFR-3-TK Inhibitor with Antilymphangiogenic, Antitumoral, and Antimetastatic Activities. MOL CANCER THER. [Article]. 2012;11(8):1637-49.

164. Shimizu Y, Shibata R, Shintani S, Ishii M, Murohara T. Therapeutic Lymphangiogenesis With Implantation of Adipose-Derived Regenerative Cells. J AM HEART ASSOC. [Article]. 2012;1(e0008774).

165. Xin X, Majumder M, Girish GV, Mohindra V, Maruyama T, Lala PK. Targeting COX-2 and EP4 to control tumor growth, angiogenesis, lymphangiogenesis and metastasis to the lungs and lymph nodes in a breast cancer model. LAB INVEST. [Article]. 2012;92(8):1115-28.

166. Larrieu-Lahargue F, Welm AL, Bouchecareilh M, Alitalo K, Li DY, Bikfalvi A, et al. Blocking Fibroblast Growth Factor Receptor Signaling Inhibits Tumor Growth, Lymphangiogenesis, and Metastasis. PLOS ONE. [Article]. 2012;7(e395406).

167. Zhang F, Niu G, Lin X, Jacobson O, Ma Y, Eden HS, et al. Imaging tumor-induced sentinel lymph node lymphangiogenesis with LyP-1 peptide. AMINO ACIDS. [Article]. 2012;42(6):2343-51.

168. Cai X, Ma S, Gu M, Zu C, Qu W, Zheng X. Survivin regulates the expression of VEGF-C in lymphatic metastasis of breast cancer. DIAGN PATHOL. [Article]. 2012;7(52).

169. Wang C, Jedlicka P, Patrick AN, Micalizzi DS, Lemmer KC, Deitsch E, et al. SIX1 induces lymphangiogenesis and metastasis via upregulation of VEGF-C in mouse models of breast cancer. J CLIN INVEST. [Article]. 2012;122(5):1895-906.

170. Majumder M, Tutunea-Fatan E, Xin X, Rodriguez-Torres M, Torres-Garcia J, Wiebe R, et al. Co-Expression of alpha 9 beta 1 Integrin and VEGF-D Confers Lymphatic Metastatic Ability to a Human Breast Cancer Cell Line MDA-MB-468LN. PLOS ONE. [Article]. 2012;7(e350944).

171. Kandemir NO, Barut F, Bektas S, Ozdamar SO. Can Lymphatic Vascular Density Be Used in Determining Metastatic Spreading Potential of Tumor in Invasive Ductal Carcinomas? PATHOL ONCOL RES. [Article]. 2012;18(2):253-62.

172. Wang Z, Wu J, Li G, Zhang X, Tong M, Wu Z, et al. Lymphangiogenesis and biological behavior in pancreatic carcinoma and other pancreatic tumors. MOL MED REP. [Article]. 2012;5(4):959-63.

173. Zampell JC, Yan A, Avraham T, Daluvoy S, Weitman ES, Mehrara BJ. HIF-1 alpha coordinates lymphangiogenesis during wound healing and in response to inflammation. FASEB J. [Article]. 2012;26(3):1027-39.

174. Acs G, Paragh G, Rakosy Z, Laronga C, Zhang PJ. The extent of retraction clefts correlates with lymphatic vessel density and VEGF-C expression and predicts nodal metastasis and poor prognosis in early-stage breast carcinoma. MODERN PATHOL. [Article]. 2012;25(2):163-77.

175. Nagahashi M, Ramachandran S, Kim EY, Allegood JC, Rashid OM, Yamada A, et al. Sphingosine-1-Phosphate Produced by Sphingosine Kinase 1 Promotes Breast Cancer Progression by Stimulating Angiogenesis and Lymphangiogenesis. CANCER RES. [Article]. 2012;72(3):726-35.

176. Wu Q, She H, Liang J, Huang Y, Yang Q, Yang Q, et al. Expression and clinical significance of extracellular matrix protein 1 and vascular endothelial growth factor-C in lymphatic metastasis of human breast cancer. BMC CANCER. [Article]. 2012;12(47).

177. Aoyagi T, Nagahashi M, Yamada A, Takabe K. The Role of Sphingosine-1-Phosphate in Breast Cancer Tumor-Induced Lymphangiogenesis. LYMPHAT RES BIOL. [Article]. 2012;10(3):97-106.

178. Lee H, Lim C, Cheong Y, Singh M, Gam L. Comparison of Protein Expression Profiles of Different Stages of Lymph Nodes Metastasis in Breast Cancer. INT J BIOL SCI. [Article]. 2012;8(3):353-62.

179. Li X, Dang X, Sun X. Expression of survivin and VEGF-C in breast cancer tissue and its relation to lymphatic metastasis. EUR J GYNAECOL ONCOL. [Article]. 2012;33(2):178-82.

180. Sakurai A, Doci C, Gutkind JS. Semaphorin signaling in angiogenesis, lymphangiogenesis and cancer. CELL RES. [Review]. 2012;22(1):23-32.

181. Wu W, Fauzee NJS, Wang Y. 5-Aminoisoquinolinone Reduces the Expression of Vascular Endothelial Growth Factor-C via the Nuclear Factor-kappa B Signaling Pathway in CT26 Cells. ASIAN PACIFIC JOURNAL OF CANCER PREVENTION. [Article]. 2012;13(3):991-4.

182. Zampell JC, Avraham T, Yoder N, Fort N, Yan A, Weitman ES, et al. Lymphatic function is regulated by a coordinated expression of lymphangiogenic and anti-lymphangiogenic cytokines. AM J PHYSIOL-CELL PH. [Article]. 2012;302(2):C392-404.

183. Cao Z, Shang B, Zhang G, Miele L, Sarkar FH, Wang Z, et al. Tumor cell-mediated neovascularization and lymphangiogenesis contrive tumor progression and cancer metastasis. BBA-REV CANCER. [Review]. 2013;1836(2):273-86.

184. Thiele W, Rothley M, Teller N, Jung N, Bulat B, Plaumann D, et al. Delphinidin is a novel inhibitor of lymphangiogenesis but promotes mammary tumor growth and metastasis formation in syngeneic experimental rats. CARCINOGENESIS. [Article]. 2013;34(12):2804-13.

185. Yang H, Zou LG, Zhang S, Gong MF, Zhang D, Qi YY, et al. Feasibility of MR imaging in evaluating breast cancer lymphangiogenesis using Polyethylene glycol-GoldMag nanoparticles. CLIN RADIOL. [Article]. 2013;68(12):1233-40.

186. Yao G, He P, Chen L, Hu X, Gu F, Ye C. MT1-MMP in breast cancer: induction of VEGF-C correlates with metastasis and poor prognosis. CANCER CELL INT. [Article]. 2013;13(98).

187. Mimori K, Shinden Y, Eguchi H, Sudo T, Sugimachi K. Biological and molecular aspects of lymph node metastasis in gastro-intestinal cancer. INT J CLIN ONCOL. [Review]. 2013;18(5):762-5.

188. Shibata M, Shibata E, Morimoto J, Harada-Shiba M. Therapy with siRNA for Vegf-c but Not for Vegf-d Suppresses Wide-spectrum Organ Metastasis in an Immunocompetent Xenograft Model of Metastatic Mammary Cancer. ANTICANCER RES. [Article]. 2013;33(10):4237-47.

189. Semenza GL. Cancer-stromal cell interactions mediated by hypoxia-inducible factors promote angiogenesis, lymphangiogenesis, and metastasis. ONCOGENE. [Review]. 2013;32(35):4057-63.

190. Clemente M, Rodriguez Sanchez-Archidona A, Sardon D, Diez L, Martin-Ruiz A, Caceres S, et al. Different role of COX-2 and angiogenesis in canine inflammatory and non-inflammatory mammary cancer. VET J. [Article]. 2013;197(2):427-32.

191. Matsumoto M, Roufail S, Inder R, Caesar C, Karnezis T, Shayan R, et al. Signaling for lymphangiogenesis via VEGFR-3 is required for the early events of metastasis. CLIN EXP METASTAS. [Article]. 2013;30(6):819-32.

192. Morgillo F, De Vita F, Antoniol G, Orditura M, Auriemma PP, Diadema MR, et al. Serum insulin-like growth factor 1 correlates with the risk of nodal metastasis in endocrine-positive breast cancer. CURR ONCOL. [Article]. 2013;20(4):E283-8.

193. Majumder M, Xin X, Lala PK. A practical and sensitive method of quantitating lymphangiogenesis in vivo. LAB INVEST. [Article]. 2013;93(7):779-91.

194. Frewer NC, Ye L, Sun P, Owen S, Ji K, Frewer KA, et al. Potential implication of IL-24 in lymphangiogenesis of human breast cancer. INT J MOL MED. [Article]. 2013;31(5):1097-104.

195. Sleeckx N, Van Brantegem L, Fransen E, Van den Eynden G, Casteleyn C, Kroeze EV, et al. Evaluation of Immunohistochemical Markers of Lymphatic and Blood Vessels in Canine Mammary Tumours. J COMP PATHOL. [Article]. 2013;148(4):307-17.

196. Zhang X, Wang Z, Wang Z, Zhang Y, Jia Q, Wu L, et al. Impact of acetylsalicylic acid on tumor angiogenesis and lymphangiogenesis through inhibition of VEGF signaling in a murine sarcoma model. ONCOL REP. [Article]. 2013;29(5):1907-13.

197. Chen Y, Yan J, Yuan Z, Yu S, Yang C, Wang Z, et al. A meta-analysis of the relationship between lymphatic microvessel density and clinicopathological parameters in breast cancer. B CANCER. [Article]. 2013;100(3):E1-10.

198. Raica M, Cimpean AM, Ceausu R, Ribatti D, Gaje P. Interplay between Mast Cells and Lymphatic Vessels in Different Molecular Types of Breast Cancer. ANTICANCER RES. [Article]. 2013;33(3):957-63.

199. Shayan R, Inder R, Karnezis T, Caesar C, Paavonen K, Ashton MW, et al. Tumor location and nature of lymphatic vessels are key determinants of cancer metastasis. CLIN EXP METASTAS. [Article]. 2013;30(3):345-56.

200. Lee E, Koskimaki JE, Pandey NB, Popel AS. Inhibition of Lymphangiogenesis and Angiogenesis in Breast Tumor Xenografts and Lymph Nodes by a Peptide Derived from Transmembrane Protein 45A. NEOPLASIA. [Article]. 2013;15(2):112-72.

201. Chien M, Lee L, Hsiao M, Wei L, Chen C, Lai T, et al. Inhibition of Metastatic Potential in Breast Carcinoma In Vivo and In Vitro through Targeting VEGFRs and FGFRs. EVID-BASED COMPL ALT. [Article]. 2013;2013(718380).

202. Choi I, Lee YS, Chung HK, Choi D, Ecoiffier T, Lee HN, et al. Interleukin-8 reduces post-surgical lymphedema formation by promoting lymphatic vessel regeneration. ANGIOGENESIS. [Article]. 2013;16(1):29-44.

203. Ciobanu M, Eremia IA, Craitoiu S, Margaritescu C, Stepan A, Patrascu V, et al. Lymphatic microvessels density, VEGF-C, and VEGFR-3 expression in 25 cases of breast invasive lobular carcinoma. ROM J MORPHOL EMBRYO. [Article]. 2013;54(4):925-34.

204. Kanngurn S, Thongsuksai P, Chewatanakornkul S. Chalkley Microvessel but not Lymphatic Vessel Density Correlates with Axillary Lymph Node Metastasis in Primary Breast Cancers. ASIAN PACIFIC JOURNAL OF CANCER PREVENTION. [Article]. 2013;14(1):583-7.

205. Pula B, Wojnar A, Witkiewicz W, Dziegiel P, Podhorska-Okolow M. Podoplanin expression in cancer-associated fibroblasts correlates with VEGF-C expression in cancer cells of invasive ductal breast carcinoma. NEOPLASMA. [Article]. 2013;60(5):516-24.

206. Widodo I, Ferronika P, Harijadi A, Triningsih FXE, Utoro T, Soeripto. Clinicopathological Significance of Lymphangiogenesis and Tumor Lymphovascular Invasion in Indonesian Breast Cancers. ASIAN PACIFIC JOURNAL OF CANCER PREVENTION. [Article]. 2013;14(2):997-1001.

207. Caprara V, Scappa S, Garrafa E, Di Castro V, Rosano L, Bagnato A, et al. Endothelin-1 regulates hypoxia-inducible factor-1 alpha and-2 alpha stability through prolyl hydroxylase domain 2 inhibition in human lymphatic endothelial cells. LIFE SCI. [Article]. 2014;118(2):185-90.

208. Lee E, Lee SJ, Koskimaki JE, Han Z, Pandey NB, Popel AS. Inhibition of breast cancer growth and metastasis by a biomimetic peptide. SCI REP-UK. [Article]. 2014;4(7139).

209. Astin JW, Jamieson SMF, Eng TCY, Flores MV, Misa JP, Chien A, et al. An In Vivo Antilymphatic Screen in Zebrafish Identifies Novel Inhibitors of Mammalian Lymphangiogenesis and Lymphatic-Mediated Metastasis. MOL CANCER THER. [Article]. 2014;13(10):2450-62.

210. Lee E, Fertig EJ, Jin K, Sukumar S, Pandey NB, Popel AS. Breast cancer cells condition lymphatic endothelial cells within pre-metastatic niches to promote metastasis. NAT COMMUN. [Article]. 2014;5(4715).

211. Lyons TR, Borges VF, Betts CB, Guo Q, Kapoor P, Martinson HA, et al. Cyclooxygenase-2-dependent lymphangiogenesis promotes nodal metastasis of postpartum breast cancer. J CLIN INVEST. [Article]. 2014;124(9):3901-12.

212. Majumder M, Xin X, Liu L, Girish GV, Lala PK. Prostaglandin E2 receptor EP4 as the common target on cancer cells and macrophages to abolish angiogenesis, lymphangiogenesis, metastasis, and stem-like cell functions. CANCER SCI. [Article]. 2014;105(9):1142-51.

213. Podgrabinska S, Skobe M. Role of lymphatic vasculature in regional and distant metastases. MICROVASC RES. [Article]. 2014;95:46-52.

214. Takabe K, Spiegel S. Export of sphingosine-1-phosphate and cancer progression. J LIPID RES. [Review]. 2014;55(9):1839-46.

215. Lee E, Pandey NB, Popel AS. Lymphatic endothelial cells support tumor growth in breast cancer. SCI REP-UK. [Article]. 2014;4(5853).

216. Camacho L, Pena L, Gonzalez Gil A, Martin-Ruiz A, Dunner S, Illera JC. Immunohistochemical Vascular Factor Expression in Canine Inflammatory Mammary Carcinoma. VET PATHOL. [Article]. 2014;51(4):737-48.

217. Nguyen J, Luk K, Vang D, Soto W, Vincent L, Robiner S, et al. Morphine stimulates cancer progression and mast cell activation and impairs survival in transgenic mice with breast cancer. BRIT J ANAESTH. [Article]. 2014;1131(SI):4-13.

218. Li S, Li Q. Cancer stem cells and tumor metastasis (Review). INT J ONCOL. [Review]. 2014;44(6):1806-12.

219. Wu Q, Yang Q, Huang Y, She H, Liang J, Yang Q, et al. Expression and Clinical Significance of Matrix Metalloproteinase-9 in Lymphatic Invasiveness and Metastasis of Breast Cancer. PLOS ONE. [Article]. 2014;9(e978045).

220. Harris NC, Achen MG. The Proteolytic Activation of Angiogenic and Lymphangiogenic Growth Factors in Cancer - Its Potential Relevance for Therapeutics and Diagnostics. CURR MED CHEM. [Article]. 2014;21(16):1821-42.

221. Wu Q, Zheng Y, Chen D, Li X, Lu C, Zhang Z. Aberrant expression of decoy receptor 3 in human breast cancer: relevance to lymphangiogenesis. J SURG RES. [Article]. 2014;188(2):459-65.

222. Yu J, Zhang X, Kuzontkoski PM, Jiang S, Zhu W, Li DY, et al. Slit2N and Robo4 regulate lymphangiogenesis through the VEGF-C/VEGFR-3 pathway. CELL COMMUN SIGNAL. [Article]. 2014;12(25).

223. Wu M, Du Y, Liu Y, He Y, Yang C, Wang W, et al. Low Molecular Weight Hyaluronan Induces Lymphangiogenesis through LYVE-1-Mediated Signaling Pathways. PLOS ONE. [Article]. 2014;9(e928573).

224. Riabov V, Gudima A, Wang N, Mickley A, Orekhov A, Kzhyshkowska J. Role of tumor assocaited macrophages in tumor angiogenesis and lymphangiogenesis. FRONT PHYSIOL. [Article]. 2014;5(75).

225. Quagliata L, Klusmeier S, Cremers N, Pytowski B, Harvey A, Pettis RJ, et al. Inhibition of VEGFR-3 activation in tumor-draining lymph nodes suppresses the outgrowth of lymph node metastases in the MT-450 syngeneic rat breast cancer model. CLIN EXP METASTAS. [Article]. 2014;31(3):351-65.

226. Stacker SA, Williams SP, Karnezis T, Shayan R, Fox SB, Achen MG. Lymphangiogenesis and lymphatic vessel remodelling in cancer. NAT REV CANCER. [Review]. 2014;14(3):159-72.

227. Gao S, Ma J, Lu C. Prognostic significance of VEGF-C immunohistochemical expression in breast cancer: a meta-analysis. TUMOR BIOLOGY. [Article]. 2014;35(2):1523-9.

228. Liang B, Li Y. Prognostic Significance of VEGF-C Expression in Patients with Breast Cancer: A Meta-Analysis. IRAN J PUBLIC HEALTH. [Review]. 2014;43(2):128-35.

229. Sleeckx N, Van Brantegem L, Van den Eynden G, Fransen E, Casteleyn C, Van Cruchten S, et al. Lymphangiogenesis in Canine Mammary Tumours: A Morphometric and Prognostic Study. J COMP PATHOL. [Article]. 2014;150(2-3):184-93.

230. Agollah GD, Wu G, Sevick-Muraca EM, Kwon S. In Vivo Lymphatic Imaging of a Human Inflammatory Breast Cancer Model. J CANCER. [Article]. 2014;5(9):774-83.

231. Genin A, Antoine M, Aractingi S, Rouzier R. Pregnancy Stimulates Tumor Angiogenesis in Breast Carcinoma. ANTICANCER RES. [Article]. 2014;34(1A):125-31.

232. Ghaffari A, Hoskin V, Szeto A, Hum M, Liaghati N, Nakatsu K, et al. A novel role for ezrin in breast cancer angio/lymphangiogenesis. BREAST CANCER RES. [Article]. 2014;16(4385).

233. Morfoisse F, Kuchnio A, Frainay C, Gomez-Brouchet A, Delisle M, Marzi S, et al. Hypoxia Induces VEGF-C Expression in Metastatic Tumor Cells via a HIF-1 alpha-Independent Translation-Mediated Mechanism. CELL REP. [Article]. 2014;6(1):155-67.

234. Nagahashi M, Takabe K, Terracina KP, Soma D, Hirose Y, Kobayashi T, et al. Sphingosine-1-Phosphate Transporters as Targets for Cancer Therapy. BIOMED RES INT. [Review]. 2014;2014(651727).

235. Niemiec JA, Adamczyk A, Ambicka A, Mucha-Malecka A, Wysocki WM, Rys J. Triple-negative, Basal Marker-expressing, and High-grade Breast Carcinomas are Characterized by High Lymphatic Vessel Density and the Expression of Podoplanin in Stromal Fibroblasts. APPL IMMUNOHISTO M M. [Article]. 2014;22(1):10-6.

236. Sahoo PK, Jana D, Mandal PK, Basak S. Effect of Lymphangiogenesis and Lymphovascular Invasion on the Survival Pattern of Breast Cancer Patients. ASIAN PACIFIC JOURNAL OF CANCER PREVENTION. [Article]. 2014;15(15):6287-93.

237. Schlereth SL, Refaian N, Iden S, Cursiefen C, Heindl LM. Impact of the Prolymphangiogenic Crosstalk in the Tumor Microenvironment on Lymphatic Cancer Metastasis. BIOMED RES INT. [Review]. 2014;2014(639058).

238. Wang C, Harrell JC, Iwanaga R, Jedlicka P, Ford HL. Vascular endothelial growth factor C promotes breast cancer progression via a novel antioxidant mechanism that involves regulation of superoxide dismutase 3. BREAST CANCER RES. [Article]. 2014;16(4625).

239. Zhang Z, Han Y, Nian Q, Chen G, Cui S, Wang X. Tumor Invasiveness, Not Lymphangiogenesis, Is Correlated with Lymph Node Metastasis and Unfavorable Prognosis in Young Breast Cancer Patients (<= 35 Years). PLOS ONE. [Article]. 2015;10(e014437612).

240. Perez D, Rohde A, Callejon G, Perez-Ruiz E, Rodrigo I, Rivas-Ruiz F, et al. Correlation between serum levels of vascular endothelial growth factor-C and sentinel lymph node status in early breast cancer. TUMOR BIOLOGY. [Article]. 2015;36(12):9285-93.

241. Wang W, Sukamtoh E, Xiao H, Zhang G. Curcumin inhibits lymphangiogenesis in vitro and in vivo. MOL NUTR FOOD RES. [Article]. 2015;59(12):2345-54.

242. Wahal SP, Goel MM, Mehrotra R. Lymphatic vessel assessment by podoplanin (D2-40) immunohistochemistry in breast cancer. J CANCER RES THER. [Article]. 2015;11(4):798-804.

243. Luczynska E, Niemiec J, Ambicka A, Adamczyk A, Walasek T, Rys J, et al. CORRELATION BETWEEN BLOOD AND LYMPHATIC VESSEL DENSITY AND RESULTS OF CONTRAST-ENHANCED SPECTRAL MAMMOGRAPHY. POL J PATHOL. [Article]. 2015;66(3):310-22.

244. Rahman M, Mohammed S. Breast cancer metastasis and the lymphatic system. ONCOL LETT. [Review]. 2015;10(3):1233-9.

245. Xu Y, Lu W, Yang P, Peng W, Wang C, Li M, et al. A small molecular agent YL529 inhibits VEGF-D-induced lymphangiogenesis and metastasis in preclinical tumor models in addition to its known antitumor activities. BMC CANCER. [Article]. 2015;15(525).

246. Bielenberg DR, Zetter BR. The Contribution of Angiogenesis to the Process of Metastasis. CANCER J. [Review]. 2015;21(4):267-73.

247. Visuri MT, Honkonen KM, Hartiala P, Tervala TV, Halonen PJ, Junkkari H, et al. VEGF-C and VEGF-C156S in the pro-lymphangiogenic growth factor therapy of lymphedema: a large animal study. ANGIOGENESIS. [Article]. 2015;18(3):313-26.

248. Mumblat Y, Kessler O, Ilan N, Neufeld G. Full-Length Semaphorin-3C Is an Inhibitor of Tumor Lymphangiogenesis and Metastasis. CANCER RES. [Article]. 2015;75(11):2177-86.

249. Wang C, Tsai S. The non-canonical role of vascular endothelial growth factor-C axis in cancer progression. EXP BIOL MED. [Article]. 2015;240(6):718-24.

250. Karpinich NO, Caron KM. Gap Junction Coupling Is Required for Tumor Cell Migration Through Lymphatic Endothelium. ARTERIOSCL THROM VAS. [Article]. 2015;35(5):1147-55.

251. Li S, Li Q. Cancer stem cells, lymphangiogenesis, and lymphatic metastasis. CANCER LETT. [Review]. 2015;357(2):438-47.

252. Tutunea-Fatan E, Majumder M, Xin X, Lala PK. The role of CCL21/CCR7 chemokine axis in breast cancer-induced lymphangiogenesis. MOL CANCER. [Article]. 2015;14(35).

253. Jitariu AA, Cimpean AM, Kundnani NR, Raica M. Platelet-derived growth factors induced lymphangiogenesis: evidence, unanswered questions and upcoming challenges. ARCH MED SCI. [Article]. 2015;11(1):57-66.

254. Niemiec J, Sas-Korczynska B, Harazin-Lechowska A, Martynow D, Adamczyk A. Lymphatic and Blood Vessels in Male Breast Cancer. ANTICANCER RES. [Article]. 2015;35(2):1041-8.

255. Aleskandarany MA, Sonbul SN, Mukherjee A, Rakha EA. Molecular Mechanisms Underlying Lymphovascular Invasion in Invasive Breast Cancer. PATHOBIOLOGY. [Review]. 2015;82(3-4):113-23.

256. Lv L, Ma R, Yang F, Zhang X, Huang D. Lymphangiogenesis in breast cancer is associated with non-sentinel lymph node metastases in sentinel node positive patients. INT J CLIN EXP PATHO. [Article]. 2015;8(9):11171-7.

257. Shen S, Zhong S, Wang C, Huang W. Correlation of lymphovascular invasion with clinicopathological factors in invasive breast cancer: a meta-analysis. INT J CLIN EXP MED. [Article]. 2015;8(10):17789-95.

258. Varney ML, Singh RK. VEGF-C-VEGFR3/Flt4 axis regulates mammary tumor growth and metastasis in an autocrine manner. AM J CANCER RES. [Article]. 2015;5(2):616-28.

259. Zankov DP, Ogita H. Actin-Tethered Junctional Complexes in Angiogenesis and Lymphangiogenesis in Association with Vascular Endothelial Growth Factor. BIOMED RES INT. [Review]. 2015;2015(314178).

260. Li J, Chen Y, Zhang L, Xing L, Xu H, Wang Y, et al. Total saponins of panaxnotoginseng promotes lymphangiogenesis by activation VEGF-C expression of lymphatic endothelial cells. J ETHNOPHARMACOL. [Article]. 2016;193:293-302.

261. Zhu C, Qi X, Zhou X, Nie X, Gui Y. Sulfatase 2 facilitates lymphangiogenesis in breast cancer by regulating VEGF-D. ONCOL REP. [Article]. 2016;36(6):3161-71.

262. Martinez-Iglesias O, Olmeda D, Alonso-Merino E, Gomez-Rey S, Gonzalez-Lopez AM, Luengo E, et al. The nuclear corepressor 1 and the thyroid hormone receptor beta suppress breast tumor lymphangiogenesis. ONCOTARGET. [Article]. 2016;7(48):79871-84.

263. Borges VF, Elder AM, Lyons TR. Deciphering Pro-Lymphangiogenic Programs during Mammary Involution and Postpartum Breast Cancer. FRONT ONCOL. [Review]. 2016;6(227).

264. Strassburg S, Torio-Padron N, Finkenzeller G, Frankenschmidt A, Stark GB. Adipose-Derived Stem Cells Support Lymphangiogenic Parameters In Vitro. J CELL BIOCHEM. [Article]. 2016;117(11):2620-9.

265. Alishekevitz D, Gingis-Velitski S, Kaidar-Person O, Gutter-Kapon L, Scherer SD, Raviv Z, et al. Macrophage-Induced Lymphangiogenesis and Metastasis following Paclitaxel Chemotherapy Is Regulated by VEGFR3. CELL REP. [Article]. 2016;17(5):1344-56.

266. Paduch R. The role of lymphangiogenesis and angiogenesis in tumor metastasis. CELL ONCOL. [Review]. 2016;39(5):397-410.

267. Krasnick BA, Nathanson SD, Arbabi CN, Chitale DA, Peterson EL. The predictive value of increased sentinel lymph node volume in breast cancer. SURG ONCOL. [Article; Proceedings Paper]. 2016;25(3):321-5.

268. Panagiotopoulos N, Lagoudianakis E, Pappas A, Filis K, Salemis N, Manouras A, et al. Lymphovascular infiltration in the tumor bed is a useful marker of biological behavior in breast cancer. J BUON. [Article]. 2016;21(5):1082-9.

269. Tsuchida J, Nagahashi M, Nakajima M, Moro K, Tatsuda K, Ramanathan R, et al. Breast cancer sphingosine-1-phosphate is associated with phospho-sphingosine kinase 1 and lymphatic metastasis. J SURG RES. [Article]. 2016;205(1):85-94.

270. Cai Y, Zhang J, Lao X, Jiang H, Yu Y, Deng Y, et al. Construction of a disulfide-stabilized diabody against fibroblast growth factor-2 and the inhibition activity in targeting breast cancer. CANCER SCI. [Article]. 2016;107(8):1141-50.

271. Jing Q, Wang Y, Liu H, Deng X, Jiang L, Liu R, et al. FGFs: crucial factors that regulate tumour initiation andprogression. CELL PROLIFERAT. [Review]. 2016;49(4):438-47.

272. Steinskog ESS, Sagstad SJ, Wagner M, Karlsen TV, Yang N, Markhus CE, et al. Impaired lymphatic function accelerates cancer growth. ONCOTARGET. [Article]. 2016;7(29):45789-802.

273. Jung M, Oeren B, Mora J, Mertens C, Dziumbla S, Popp R, et al. Lipocalin 2 from macrophages stimulated by tumor cell-derived sphingosine-1-phosphate promotes lymphangiogenesis and tumor metastasis. SCI SIGNAL. [Article]. 2016;9(ra64434).

274. Cha YJ, Youk JH, Kim BG, Jung WH, Cho NH. Lymphangiogenesis in Breast Cancer Correlates with Matrix Stiffness on Shear-Wave Elastography. YONSEI MED J. [Article]. 2016;57(3):599-605.

275. Dieterich LC, Detmar M. Tumor lymphangiogenesis and new drug development. ADV DRUG DELIVER REV. [Review]. 2016;99(B):148-60.

276. Yoshimatsu Y, Miyazaki H, Watabe T. Roles of signaling and transcriptional networks in pathological lymphangiogenesis. ADV DRUG DELIVER REV. [Review]. 2016;99(B):161-71.

277. Maeng Y, Aguilar B, Choi S, Kim EK. Inhibition of TGFBIp expression reduces lymphangiogenesis and tumor metastasis. ONCOGENE. [Article]. 2016;35(2):196-205.

278. Bron S, Henry L, Faes-van'T Hull E, Turrini R, Vanhecke D, Guex N, et al. TIE-2-expressing monocytes are lymphangiogenic and associate specifically with lymphatics of human breast cancer. ONCOIMMUNOLOGY. [Article]. 2016;5(e10738822).

279. Donizy P, Kaczorowski M, Halon A, Leskiewicz M, Matkowski R. Lymphangioinvasion in routine H&E staining is strongly associated with poor clinical outcome in lymph node-negative cutaneous melanoma patients. FOLIA HISTOCHEM CYTO. [Article]. 2016;54(3):126-33.

280. Stachura J, Wachowska M, Kilarski WW, Guc E, Golab J, Muchowicz A. The dual role of tumor lymphatic vessels in dissemination of metastases and immune response development. ONCOIMMUNOLOGY. [Review]. 2016;5(e11822787).

281. Chen Y, Liu Y, Wang Y, Li W, Wang X, Liu X, et al. Quantification of STAT3 and VEGF expression for molecular diagnosis of lymph node metastasis in breast cancer. MEDICINE. [Article]. 2017;96(e848845).

282. You K, Su F, Liu L, Lv X, Zhang J, Zhang Y, et al. SCARA5 plays a critical role in the progression and metastasis of breast cancer by inactivating the ERK1/2, STAT3, and AKT signaling pathways. MOL CELL BIOCHEM. [Article]. 2017;435(1-2):47-58.

283. Choi JU, Chung SW, Al-Hilal TA, Alam F, Park J, Mahmud F, et al. A heparin conjugate, LHbisD4, inhibits lymphangiogenesis and attenuates lymph node metastasis by blocking VEGF-C signaling pathway. BIOMATERIALS. [Article]. 2017;139:56-66.

284. Muchowicz A, Wachowska M, Stachura J, Tonecka K, Gabrysiak M, Wolosz D, et al. Inhibition of lymphangiogenesis impairs antitumour effects of photodynamic therapy and checkpoint inhibitors in mice. EUR J CANCER. [Article]. 2017;83:19-27.

285. Weichand B, Popp R, Dziumbla S, Mora J, Strack E, Elwakeel E, et al. S1PR1 on tumor-associated macrophages promotes lymphangiogenesis and metastasis via NLRP3/IL-1 beta. J EXP MED. [Article]. 2017;214(9):2695-713.

286. Ran S, Wilber A. Novel role of immature myeloid cells in formation of new lymphatic vessels associated with inflammation and tumors. J LEUKOCYTE BIOL. [Review]. 2017;102(2):253-63.

287. Garcia-Caballero M, Paupert J, Blacher S, Van de Velde M, Rodriguez Quesada A, Angel Medina M, et al. Targeting VEGFR-3/-2 signaling pathways with AD0157: a potential strategy against tumor-associated lymphangiogenesis and lymphatic metastases. J HEMATOL ONCOL. [Article]. 2017;10(122).

288. Maiborodin IV, Kozyakov AE, Babayants EV, Krasil'Nikov SE. Features of Blood Supply to Axillary Lymph Nodes in Breast Cancer Patients. B EXP BIOL MED+. [Article]. 2017;163(1):82-6.

289. Keser SH, Kandemir NO, Ece D, Gecmen GG, Gul AE, Barisik NO, et al. Relationship of mast cell density with lymphangiogenesis and prognostic parameters in breast carcinoma. KAOHSIUNG J MED SCI. [Article]. 2017;33(4):171-80.

290. Oh N, Park J, Park J, Kim K, Lee DR, Park K. The role of ELK3 to regulate peritumoral lymphangiogenesis and VEGF-C production in triple negative breast cancer cells. BIOCHEM BIOPH RES CO. [Article]. 2017;484(4):896-902.

291. Americo MG, Freire Soares Marques YM, El Abras Ankha MDV, Do Prado RF, Carvalho YR. Correlation of intratumoral lymphatic microvessel density, vascular endothelial growth factor C and cell proliferation in salivary gland tumors. MED MOL MORPHOL. [Article]. 2017;50(1):17-24.

292. Eroglu A, Ersoz C, Karasoy D, Sak S. Vascular endothelial growth factor (VEGF)-C, VEGF-D, VEGFR-3 and D2-40 expressions in primary breast cancer: Association with lymph node metastasis. ADV CLIN EXP MED. [Article]. 2017;26(2):245-9.

293. Wang S, Chang JS, Hsiao J, Yen Y, Jiang SS, Liu S, et al. Tumour cell-derived WNT5B modulates in vitro lymphangiogenesis via induction of partial endothelial-mesenchymal transition of lymphatic endothelial cells. ONCOGENE. [Article]. 2017;36(11):1503-15.

294. Zhang S, Zhang D, Yi S, Gong M, Lu C, Cai Y, et al. The relationship of lymphatic vessel density, lymphovascular invasion, and lymph node metastasis in breast cancer: a systematic review and meta-analysis. ONCOTARGET. [Review]. 2017;8(2):2863-73.

295. Zhang S, Yi S, Zhang D, Gong M, Cai Y, Zou L. Intratumoral and peritumoral lymphatic vessel density both correlate with lymph node metastasis in breast cancer. SCI REP-UK. [Article]. 2017;7(40364).

296. Nandi P, Girish GV, Majumder M, Xin X, Tutunea-Fatan E, Lala PK. PGE2 promotes breast cancer-associated lymphangiogenesis by activation of EP4 receptor on lymphatic endothelial cells. BMC CANCER. [Article]. 2017;17(11).

297. Niemiec JA, Adamczyk A, Ambicka A, Mucha-Malecka A, Wysocki WM, Biesaga B, et al. Prognostic role of lymphatic vessel density and lymphovascular invasion in chemotherapy-naive and chemotherapy-treated patients with invasive breast cancer. AM J TRANSL RES. [Article]. 2017;9(3):1435-47.

298. Tsuchida J, Nagahashi M, Takabe K, Wakai T. Clinical Impact of Sphingosine-1-Phosphate in Breast Cancer. MEDIAT INFLAMM. [Review]. 2017;2017(2076239).

299. Wei J, Yang J, Liu D, Wu M, Qiao L, Wang J, et al. Tumor-associated Lymphatic Endothelial Cells Promote Lymphatic Metastasis By Highly Expressing and Secreting SEMA4C. CLIN CANCER RES. [Article]. 2017;23(1):214-24.

300. Chen Y, Keskin D, Sugimoto H, Kanasaki K, Phillips PE, Bizarro L, et al. Podoplanin(+) tumor lymphatics are rate limiting for breast cancer metastasis. PLOS BIOL. [Article]. 2018;16(e200590712).

301. Chatterjee G, Pai T, Hardiman T, Avery-Kiejda K, Scott RJ, Spencer J, et al. Molecular patterns of cancer colonisation in lymph nodes of breast cancer patients. BREAST CANCER RES. [Review]. 2018;20(143).

302. Elder AM, Tamburini BAJ, Crump LS, Black SA, Wessells VM, Schedin PJ, et al. Semaphorin 7A Promotes Macrophage-Mediated Lymphatic Remodeling during Postpartum Mammary Gland Involution and in Breast Cancer. CANCER RES. [Article]. 2018;78(22):6473-85.

303. Yamakawa M, Doh SJ, Santosa SM, Montana M, Qin EC, Kong H, et al. Potential lymphangiogenesis therapies: Learning from current antiangiogenesis therapies-A review. MED RES REV. [Review]. 2018;38(6):1769-98.

304. Singh PP, Sood NK, Gupta K, Narang D. Intratumoural and peritumoural lymphangiogenesis in canine mammary tumour linked to tumour spread and poor survival. CURR SCI INDIA. [Article]. 2018;115(7):1312-9.

305. Hu X, Luo J. Heterogeneity of tumor lymphangiogenesis: Progress and prospects. CANCER SCI. [Review]. 2018;109(10):3005-12.

306. Pascale F, Bedouet L, Fazel A, Namur J, Ghegediban SH, Cornil IS, et al. Lymphatic Transport and Lymph Node Location of Microspheres Subcutaneously Injected in the Vicinity of Tumors in a Rabbit Model of Breast Cancer. PHARM RES-DORDR. [Article]. 2018;35(19110).

307. Xiong Y, Liu Z, Zhao X, Ruan S, Zhang X, Wang S, et al. CPT1A regulates breast cancer-associated lymphangiogenesis via VEGF signaling. BIOMED PHARMACOTHER. [Article]. 2018;106:1-7.

308. Lala PK, Nandi P, Majumder M. Roles of prostaglandins in tumor-associated lymphangiogenesis with special reference to breast cancer. CANCER METAST REV. [Review]. 2018;37(2-3SI):369-84.

309. Harris AR, Perez MJ, Munson JM. Docetaxel facilitates lymphatic-tumor crosstalk to promote lymphangiogenesis and cancer progression. BMC CANCER. [Article]. 2018;18(718).

310. Fristiohady A, Milovanovic D, Krieger S, Huttary N, Chi HN, Basilio J, et al. 12(S)-HETE induces lymph endothelial cell retraction in vitro by upregulation of SOX18. INT J ONCOL. [Article]. 2018;53(1):307-16.

311. Jin K, Pandey NB, Popel AS. Simultaneous blockade of IL-6 and CCL5 signaling for synergistic inhibition of triple-negative breast cancer growth and metastasis. BREAST CANCER RES. [Article]. 2018;20(54).

312. Mitrofanova I, Zavyalova M, Riabov V, Cherdyntseva N, Kzhyshkowska J. The effect of neoadjuvant chemotherapy on the correlation of tumor-associated macrophages with CD31 and LYVE-1. IMMUNOBIOLOGY. [Article]. 2018;223(6-7):449-59.

313. Guleria P, Srinivas V, Basannar D, Dutta V. Comparison of lymphangiogenesis, lymphatic invasion, and axillary lymph node metastasis in breast carcinoma. INDIAN J PATHOL MICR. [Article]. 2018;61(2):176-80.

314. Majumder M, Nandi P, Omar A, Ugwuagbo KC, Lala PK. EP4 as a Therapeutic Target for Aggressive Human Breast Cancer. INT J MOL SCI. [Review]. 2018;19(10194).

315. Wang T, Zheng L, Wang Q, Hu Y. Emerging roles and mechanisms of FOXC2 in cancer. CLIN CHIM ACTA. [Review]. 2018;479:84-93.

316. Brown M, Assen FP, Leithner A, Abe J, Schachner H, Asfour G, et al. Lymph node blood vessels provide exit routes for metastatic tumor cell dissemination in mice. SCIENCE. [Article]. 2018;359(6382SI):1408.

317. Agarwal S, Singh A, Bagga PK. Immunohistochemical evaluation of lymphovascular invasion in carcinoma breast with CD34 and D2-40 and its correlation with other prognostic markers. INDIAN J PATHOL MICR. [Article]. 2018;61(1):39-44.

318. Wang Q, He R, Yang F, Kang L, Li X, Fu L, et al. FOXF2 deficiency permits basal-like breast cancer cells to form lymphangiogenic mimicry by enhancing the response of VEGF-C/VEGFR3 signaling pathway. CANCER LETT. [Article]. 2018;420:116-26.

319. Zamora A, Alves M, Chollet C, Therville N, Fougeray T, Tatin F, et al. Paclitaxel induces lymphatic endothelial cells autophagy to promote metastasis. CELL DEATH DIS. [Article]. 2019;10(956).

320. Bieniasz-Krzywiec P, Martin-Perez R, Ehling M, Garcia-Caballero M, Pinioti S, Pretto S, et al. Podoplanin-Expressing Macrophages Promote Lymphangiogenesis and Lymphoinvasion in Breast Cancer. CELL METAB. [Article]. 2019;30(5):917-36.

321. Ginter PS, Karagiannis GS, Entenberg D, Lin Y, Condeelis J, Jones JG, et al. Tumor Microenvironment of Metastasis (TMEM) Doorways Are Restricted to the Blood Vessel Endothelium in Both Primary Breast Cancers and Their Lymph Node Metastases. CANCERS. [Article]. 2019;11(150710).

322. Zajkowska M, Lubowicka E, Fiedorowicz W, Szmitkowski M, Jamiolkowski J, Lawicki S. Human Plasma Levels of VEGF-A, VEGF-C, VEGF-D, their Soluble Receptor-VEGFR-2 and Applicability of these Parameters as Tumor Markers in the Diagnostics of Breast Cancer. PATHOL ONCOL RES. [Article]. 2019;25(4):1477-86.

323. Kato S, Shirai Y, Sakamoto M, Mori S, Kodama T. Use of a Lymphatic Drug Delivery System and Sonoporation to Target Malignant Metastatic Breast Cancer Cells Proliferating in the Marginal Sinuses. SCI REP-UK. [Article]. 2019;9(13242).

324. Hunter S, Nault B, Ugwuagbo KC, Maiti S, Majumder M. Mir526b and Mir655 Promote Tumour Associated Angiogenesis and Lymphangiogenesis in Breast Cancer. CANCERS. [Article]. 2019;11(9387).

325. Wang C, Xu S, Tian Y, Du A, Hou O, Liu J, et al. Lysyl Oxidase-Like Protein 2 Promotes Tumor Lymphangiogenesis and Lymph Node Metastasis in Breast Cancer. NEOPLASIA. [Article]. 2019;21(4):413-27.

326. Wang J, Ma Y, Yang J, Jin L, Gao Z, Xue L, et al. Fucoxanthin inhibits tumour-related lymphangiogenesis and growth of breast cancer. J CELL MOL MED. [Article]. 2019;23(3):2219-29.

327. Mazzone M, Bergers G. Regulation of Blood and Lymphatic Vessels by Immune Cells in Tumors and Metastasis. In: Nelson MT, Walsh K, ^editors. Annual Review of Physiology. PALO ALTO: ANNUAL REVIEWS; 2019. p. 535-60.

328. Zhao Z, Li L, Du P, Ma L, Zhang W, Zheng L, et al. Transcriptional Downregulation of miR-4306 serves as a New Therapeutic Target for Triple Negative Breast Cancer. THERANOSTICS. [Article]. 2019;9(5):1401-16.

329. Grimm D, Bauer J, Wise P, Krueger M, Simonsen U, Wehland M, et al. The role of SOX family members in solid tumours and metastasis. SEMIN CANCER BIOL. [Review]. 2020;67(1):122-53.

330. Md Yusof K, Rosli R, Abdullah M, Avery-Kiejda KA. The Roles of Non-Coding RNAs in Tumor-Associated Lymphangiogenesis. CANCERS. [Review]. 2020;12(329011).

331. Korbecki J, Grochans S, Gutowska I, Barczak K, Baranowska-Bosiacka I. CC Chemokines in a Tumor: A Review of Pro-Cancer and Anti-Cancer Properties of Receptors CCR5, CCR6, CCR7, CCR8, CCR9, and CCR10 Ligands. INT J MOL SCI. [Review]. 2020;21(761920).

332. Dumond A, Pages G. Neuropilins, as Relevant Oncology Target: Their Role in the Tumoral Microenvironment. FRONTIERS IN CELL AND DEVELOPMENTAL BIOLOGY. [Review]. 2020;8(662).

333. Esa R, Steinberg E, Dror D, Schwob O, Khajavi M, Maoz M, et al. The Role of Methionine Aminopeptidase 2 in Lymphangiogenesis. INT J MOL SCI. [Article]. 2020;21(514814).

334. Zhou H, Blevins MA, Hsu JY, Kong D, Galbraith MD, Goodspeed A, et al. Identification of a Small-Molecule Inhibitor That Disrupts the SIX1/EYA2 Complex, EMT, and Metastasis. CANCER RES. [Article]. 2020;80(12):2689-702.

335. Elder AM, Stoller AR, Black SA, Lyons TR. Macphatics and PoEMs in Postpartum Mammary Development and Tumor Progression. J MAMMARY GLAND BIOL. [Review]. 2020;25(2):103-13.

336. Baran M, Ozturk F, Canoz O, Onder GO, Yay A. The effects of apoptosis and apelin on lymph node metastasis in invasive breast carcinomas. CLIN EXP MED. [Article]. 2020;20(4):507-14.

337. Asaoka M, Patnaik SK, Zhang F, Ishikawa T, Takabe K. Lymphovascular invasion in breast cancer is associated with gene expression signatures of cell proliferation but not lymphangiogenesis or immune response. BREAST CANCER RES TR. [Article]. 2020;181(2):309-22.

338. Kesavan R, Froemel T, Zukunft S, Laban H, Geyer A, Naeem Z, et al. Cyp2c44 regulates prostaglandin synthesis, lymphangiogenesis, and metastasis in a mouse model of breast cancer. P NATL ACAD SCI USA. [Article]. 2020;117(11):5923-30.

339. Ahmadzadeh N, Robering JW, Kengelbach-Weigand A, Al-Abboodi M, Beier JP, Horch RE, et al. Human adipose-derived stem cells support lymphangiogenesis in vitro by secretion of lymphangiogenic factors. EXP CELL RES. [Article]. 2020;388(1118162).

340. Zheng S, Yang L, Zou Y, Liang J, Liu P, Gao G, et al. Long non-coding RNA HUMT hypomethylation promotes lymphangiogenesis and metastasis via activating FOXK1 transcription in triple-negative breast cancer. J HEMATOL ONCOL. [Article]. 2020;13(171).

341. Jones D. Parallels of Resistance between Angiogenesis and Lymphangiogenesis Inhibition in Cancer Therapy. CELLS-BASEL. [Review]. 2020;9(7623).

342. Ayuso JM, Gong MM, Skala MC, Harari PM, Beebe DJ. Human Tumor-Lymphatic Microfluidic Model Reveals Differential Conditioning of Lymphatic Vessels by Breast Cancer Cells. ADV HEALTHC MATER. [Article]. 2020;9(19009253).

343. Ran S, Volk-Draper L. Lymphatic Endothelial Cell Progenitors in the Tumor Microenvironment. In: Birbrair A, editor Advances in Experimental Medicine and Biology. CHAM: SPRINGER INTERNATIONAL PUBLISHING AG; 2020. p. 87-105.

344. Zajkowska M, Gacuta E, Lubowicka E, Szmitkowski M, Lawicki S. Can VEGFR-3 be a better tumor marker for breast cancer than CA 15-3? ACTA BIOCHIM POL. [Article]. 2020;67(1):25-9.

345. Ji K, Zhao Z, Sameni M, Moin K, Xu Y, Gillies RJ, et al. Modeling Tumor: Lymphatic Interactions in Lymphatic Metastasis of Triple Negative Breast Cancer. CANCERS. [Article]. 2021;13(604423).

346. Park M, Kim J, Kim T, Kim S, Park W, Ha K, et al. REDD1 is a determinant of low-dose metronomic doxorubicin-elicited endothelial cell dysfunction through downregulation of VEGFR-2/3 expression. EXP MOL MED. [Article]. 2021;53(10):1612-22.

347. Sethy C, Goutam K, Das B, Dash SR, Kundu CN. Nectin-4 promotes lymphangiogenesis and lymphatic metastasis in breast cancer by regulating CXCR4-LYVE-1 axis. VASC PHARMACOL. [Article]. 2021;140(106865).

348. Xing H, Yang X, Xu Y, Tang K, Tian Z, Chen Z, et al. Anti-tumor effects of vascular endothelial growth factor/vascular endothelial growth factor receptor binding domain-modified chimeric antigen receptor T cells. CYTOTHERAPY. [Article]. 2021;23(9):810-9.

349. Hou Q, Chen S, An Q, Li B, Fu Y, Luo Y. Extracellular Hsp90 alpha Promotes Tumor Lymphangiogenesis and Lymph Node Metastasis in Breast Cancer. INT J MOL SCI. [Article]. 2021;22(774714).

350. Liu T, Liu Q, Anaya I, Huang D, Kong W, Mille LS, et al. Investigating lymphangiogenesis in a sacrificially bioprinted volumetric model of breast tumor tissue. METHODS. [Article]. 2021;190:72-9.

351. He M, He Q, Cai X, Chen Z, Lao S, Deng H, et al. Role of lymphatic endothelial cells in the tumor microenvironment-a narrative review of recent advances. TRANSLATIONAL LUNG CANCER RESEARCH. [Review]. 2021;10(5):2252-77.

352. Wang M, Xie J, Fu Y, Zhou Y, Liu S. Silencing of cluster determinant 36 transmitted by gold nanoparticles inhibits the occurrence and progression of breast cancer by down-regulating the peroxisome proliferative activated receptor signaling pathway. MATER EXPRESS. [Article]. 2021;11(5):789-800.

353. Wang X, Liu Z, Sun J, Song X, Bian M, Wang F, et al. Inhibition of NADPH oxidase 4 attenuates lymphangiogenesis and tumor metastasis in breast cancer. FASEB J. [Article]. 2021;35(e215314).

354. De Paz Linares GA, Opperman RM, Majumder M, Lala PK. Prostaglandin E2 Receptor 4 (EP4) as a Therapeutic Target to Impede Breast Cancer-Associated Angiogenesis and Lymphangiogenesis. CANCERS. [Review]. 2021;13(9425).

355. Korbecki J, Kojder K, Kapczuk P, Kupnicka P, Gawronska-Szklarz B, Gutowska I, et al. The Effect of Hypoxia on the Expression of CXC Chemokines and CXC Chemokine Receptors-A Review of Literature. INT J MOL SCI. [Review]. 2021;22(8432).

356. Wasik A, Ratajczak-Wielgomas K, Badzinski A, Dziegiel P, Podhorska-Okolow M. The Role of Periostin in Angiogenesis and Lymphangiogenesis in Tumors. CANCERS. [Review]. 2022;14(422517).

357. Fan S, Cui Y, Li Y, Xu J, Shen Y, Huang H, et al. LncRNA CASC9 activated by STAT3 promotes the invasion of breast cancer and the formation of lymphatic vessels by enhancing H3K27ac-activated SOX4. KAOHSIUNG J MED SCI. [Article]. 2022;38(9):848-57.

358. Wu R, Gandhi S, Tokumaru Y, Asaoka M, Oshi M, Yan L, et al. Intratumoral PDGFB gene predominantly expressed in endothelial cells is associated with angiogenesis and lymphangiogenesis, but not with metastasis in breast cancer. BREAST CANCER RES TR. [Article]. 2022;195(1):17-31.

359. Azzarito G, Visentin M, Leeners B, Dubey RK. Transcriptomic and Functional Evidence for Differential Effects of MCF-7 Breast Cancer Cell-Secretome on Vascular and Lymphatic Endothelial Cell Growth. INT J MOL SCI. [Article]. 2022;23(719213).

360. Li J, Yan Z, Ma J, Chu Z, Li H, Guo J, et al. ZKSCAN5 Activates VEGFC Expression by Recruiting SETD7 to Promote the Lymphangiogenesis, Tumour Growth, and Metastasis of Breast Cancer. FRONT ONCOL. [Article]. 2022;12(875033).

361. Miller IS, Khan S, Shiels LP, Das S, O' Farrell AC, Connor K, et al. Implementing subtype-specific pre-clinical models of breast cancer to study pre-treatment aspirin effects. CANCER MED-US. [Article]. 2022;11(20):3820-36.

362. Bouleftour W, Guillot A, Magne N. The Anti-Nectin 4: A Promising Tumor Cells Target. A Systematic Review. MOL CANCER THER. [Review]. 2022;21(4):493-501.

363. Du Y, Cao M, Liu Y, He Y, Yang C, Zhang G, et al. Tumor microenvironment remodeling modulates macrophage phenotype in breast cancer lymphangiogenesis. FASEB J. [Article]. 2022;36(e222484).

364. Harris AR, Esparza S, Azimi MS, Cornelison R, Azar FN, Llaneza DC, et al. Platinum Chemotherapy Induces Lymphangiogenesis in Cancerous and Healthy Tissues That Can be Prevented With Adjuvant Anti-VEGFR3 Therapy. FRONT ONCOL. [Article]. 2022;12(801764).

365. Majima M, Hosono K, Ito Y, Amano H. Biologically active lipids in the regulation of lymphangiogenesis in disease states. PHARMACOL THERAPEUT. [Review]. 2022;232(108011).

366. Chen J, Luo B, Ma R, Luo X, Chen Y, Li Y. Lymphatic Endothelial Markers and Tumor Lymphangiogenesis Assessment in Human Breast Cancer. DIAGNOSTICS. [Article]. 2022;12(41).

367. Wu R, Sarkar J, Tokumaru Y, Takabe Y, Oshi M, Asaoka M, et al. Intratumoral lymphatic endothelial cell infiltration reflecting lymphangiogenesis is counterbalanced by immune responses and better cancer biology in the breast cancer tumor microenvironment. AM J CANCER RES. [Article]. 2022;12(2):504-20.

368. Chen S, Zhao C, Yao L, Wang L, Ma Y, Meng L, et al. Aiphanol, a multi-targeting stilbenolignan, potently suppresses mouse lymphangiogenesis and lymphatic metastasis. ACTA PHARMACOL SIN. [Article]. 2023;44(1):189-200.

369. Westhoff C, Mueller S, Jank P, Kalder M, Moll R. Nodal lymphangiogenesis and immunophenotypic variations of sinus endothelium in sentinel and non-sentinel lymph nodes of invasive breast carcinoma. PLOS ONE. [Article]. 2023;18(e02809361).
